# A *Colletotrichum fructicola* dual specificity phosphatase CfMsg5 is regulated by the CfAp1 transcription factor during oxidative stress and promotes virulence on *Camellia oleifera*

**DOI:** 10.1080/21505594.2024.2413851

**Published:** 2024-10-18

**Authors:** Yalan Gao, Shengpei Zhang, Song Sheng, He Li

**Affiliations:** a Key Laboratory of National Forestry and Grassland Administration on Control of Artificial Forest Diseases and Pests in South China, Central South University of Forestry and Technology, Changsha, China; b Hunan Provincial Key Laboratory for Control of Forest Diseases and Pests, Central South University of Forestry and Technology, Changsha, China; c Yuelushan Laboratory Non-wood Forests Variety Innovation Center, Changsha, China

**Keywords:** *Colletotrichum fructicola*, anthracnose, Msg5, Ap1, ROS, pathogenicity

## Abstract

Anthracnose, caused by *Colletotrichum* species, induces significant economic damages to crop plants annually, especially for *Camellia oleifera*. During infection, the counter-defence mechanisms of plant pathogens against ROS-mediated resistance, however, remain poorly understood. By employing Weighted Gene Co-expression Network Analysis (WGCNA), we identified ACTIVATOR PROTEIN-1 (AP-1), a bZIP transcription factor, as significant to infection. And deletion of *CfAP1* inhibited aerial hyphae formation and growth under oxidative stress. Furthermore, RNA-seq analysis post H_2_O_2_ treatment revealed 33 significantly down-regulated genes in the AP-1 deficient strain, including A12032, a dual specificity phosphatase (DSP) homologous to MSG5 from *Saccharomyces cerevisiae*. This Δ*Cfmsg5* strain showed enhanced oxidative tolerance, reduced ROS scavenging, and negative regulation of the CWI MAPK cascade under oxygen stress, suggesting its involvement in oxidative signal transduction. Importantly, we provide evidence that CfMsg5 regulates growth, endoplasmic reticulum stress, and several unfolded protein response genes upregulated in Δ*Cfmsg5*. Collectively, this study identified core components during *C. fructicola* infection and highlights a potential regulatory module involving CfAp1 and CfMsg5 in response to host ROS bursts. It provides new insights into fungal infection mechanisms and potential targets like *CfAP1* and *CfMSG5* for managing anthracnose diseases.

## Introduction

Pathogens, insects, and various abiotic stresses, such as floods, extended droughts, and high temperatures, can cause significant losses in agricultural productivity and pose a threat to global food security. The alarming escalation in both frequency and severity of various plant stresses, including heat, drought, and pathogen and pest challenges, can be attributed to the impacts of global warming and climate change [1–3]. This trend underscores the critical importance of comprehensively understanding the underlying mechanisms to enhance plant stress resistance and adaptability.

Plant pathogen infections account for roughly 15% of worldwide agricultural production losses annually, and fungi have the potential to destroy enough food to feed up to 60% of the world’s population [4]. *Camellia oleifera* Abel. (commonly known as oil tea, Theaceae) is one of four major woody oil plants that widely distributed in subtropical mountain areas of the Yangtze River basin and southern China [5,6]. The plantation area of *Ca. oleifera* reached approximately 4.4 million hectares, with an annual output of over 2.6 million tons of seeds and, as a result, oil yields of more than 0.65 million tons [7]. Surprisingly, oil tea frequently suffers from anthracnose fungal infestation, which causes 20% to 40% fruit drop and 10% to 30% seed loss annually, as well as branch dieback and even plant death [8].

*Colletotrichum* Corda is common pathogen that causes variety woody and herbaceous plant diseases in the tropics and subtropics, and can cause pre- or post-harvest diseases of some cash crops, such as strawberries, mangoes, citrus, and even some food crops. The massive reduction in crop yields has led to significant economic losses and has also affected the agricultural development in many developing countries [9]. Based on its economic and scientific importance, this genus of fungi is ranked as the eighth most important plant pathogen worldwide [10]. Since Li et al. [11] identified that the dominant epidemic pathogenicity of anthracnose in oil tea is *Colletotrichum fructicola*. Up until now, we have thoroughly examined the morphological characteristics, drug resistance, population genetic structure, and functions of numerous genes from *C. fructicola*, including CfSnf1, CfHac1, and CfGcn5. These studies have established a solid foundation for future investigations into the pathogenic processes of this organism [12–14]. Nevertheless, due to the long-term use of chemical fungicides for the control of oil tea anthracnose, the anthracnose pathogen of oil tea had developed more serious resistance in China. Therefore, it is critical to elucidate the pathogenic molecular mechanism of the anthracnose pathogen for the development of new fungicide targets.

Anthracnose symptoms include denting and necrosis of leaves, stems, flowers, and fruits, as well as stem and leaf spots and seedling wilting. Furthermore, as endophytes or saprophytes, several fungi can switch to a pathogenic lifestyle and cause disease when host plants are kept under stressful conditions or after harvest. The pathogen causes anthracnose by developing orange hyphal clasping discs on the surface or under the cortex of the host and black bristles on the discs; the genus’ clasping method is endophytic budding; and the hyphal clasps are colourless, solitary clasping, and columnar, crescent-shaped, and spindly. The mycelium that overwinters on dead host tissues in the form of fungal spores or decay is often the cause of the first infestation of *C. fructicola*, however some perennial crops, dead fruits, and leaves can also become its overwintering habitats. *C. fructicola* is primarily disseminated by hyphal clasper rain splash; environmental conditions such as temperature and humidity play an important impact in the disease’s epidemiology.

The plant perception of pathogen-associated molecular patterns triggers a plethora of cellular immune responses. One of these responses is a rapid and transient burst of reactive oxygen species (ROS) mediated by plasma membrane-localized NADPH oxidases [15]. ROS are commonly produced in plant–pathogen interactions, and as oxidative molecules, ROS can effectively inhibit pathogen infestation while also causing oxidative damage to the host’s own cells; however, ROS can also act as signalling molecules, effectively activating the host’s defence response, improving the host’s resistance to disease and limiting further pathogen expansion [15]. However, pathogen have developed a series of ROS elimination mechanisms that are involved in the elimination of host ROS, therefore suppressing ROS-mediated host defensive responses [16].

Plants evolved and progressively developed a series of sophisticated and effective defence mechanisms known as the plant innate immune system, which includes two layers to sense pathogen invasion during long-term interactions with harmful microorganisms. PTI identifies pathogen-associated molecular patterns via pattern recognition receptors in the first layer (PRRs). The immunological response is generated by PRRs on the plant cell surface recognizing PAMPs or microbial-associated molecular patterns (MAMPs) [17,18]. These MAMPs/PAMPs encompass a variety of conserved compounds that are generally produced by various microbes. The list includes substances such as fungal chitin, bacterial flagellin, lipopolysaccharide (LPS), transcriptional elongation factor (EF-Tu), and oomycete exocytosis proteins like exciton (INF1) and glycosyl hydrolase (XEG1). Upon recognition of PAMPs by PRRs, the host cell undergoes a series of intracellular physiological and biochemical transformations. These alterations are part of the plant’s defence arsenal against pathogenic fungi and include responses such as the burst of ROS, production of phytochemicals, accumulation of disease-related proteins, and changes within the plant’s defensive enzyme system. Recent research has further elucidated these mechanisms. For example, LysM receptor-like kinases in plants perceive lipo-chitooligosaccharides (LCOs) and modulate plant defence against arbuscular mycorrhizal fungi [19]. Additionally, studies on the lipopolysaccharide (LPS) O-antigen synthesis gene in *Mesorhizobium huakuii* revealed its differentiated roles in root nodule symbiotic compatibility with *Astragalus sinicus* [20]. Furthermore, research on the grapevine LysM receptor-like kinase VvLYK5-1 showed its interaction with chitin oligomers, facilitating plant defence against fungal pathogens [21]. These findings collectively enhance our understanding of plant–microbe interactions and the sophisticated defence mechanisms plants employ against diverse pathogens.

In this study, we employed Weighted Gene Co-expression Network Analysis (WGCNA) to delve deeper into the infection mechanism of *C. fructicola* on oil-tea. WGCNA is a systems biology method used for describing the correlation patterns among genes across microarray samples. It can be used to identify clusters (modules) of highly correlated genes, summarizing such clusters using the module eigengene or an intramodular hub gene, and relating modules to one another and to external sample traits [22]. This method has been widely applied in various biological contexts, including the study of plant-pathogen interactions [23], offering valuable insights into complex biological processes.

In our approach, we processed WGCNA with transcriptome data from different cultivars, leading to the identification of Activation Protein 1 (Ap1) as a core protein positively correlated with leaf lesion size. This finding aligns with previous studies that have underscored the significance of Ap1 in plant defence mechanisms. Compared to wildtype and complementary strains, the growth rate of the deletion mutant Δ*Cfap1* was significantly affected, with notable reductions in aerial mycelium, pathogenicity, and oxidative stress tolerance.

Further RNA-seq analysis of an *AP1* deletion mutant identified 33 potential downstream oxidative response genes. Among these, 3 genes were found to be defective in pathogenicity, with only CfMsg5 being associated with H_2_O_2_ stress. Intriguingly, while *MSG5* knockouts exhibit impaired growth, they demonstrate a lesser impact from H_2_O_2_ treatment compared to wildtype *C. fructicola*. This suggests that the Msg5 deletion mutant possesses enhanced oxidative tolerance. Additionally, our results indicated that CfMsg5 play a role in oxidative signal transduction, although no AP1 binding cis-regulatory element was detected in the −1 to −2000 promoter region. Collectively, our study integrates a novel regulatory model that indirectly incorporates *CfAP1* and *CfMSG5* in responses to external oxidative stress, potentially offering a foundational understanding of the mechanisms of *C. fructicola* in response to plant immune ROS burst.

## Results

### WGCNA to identify hub genes during infection

**Figure 1.**
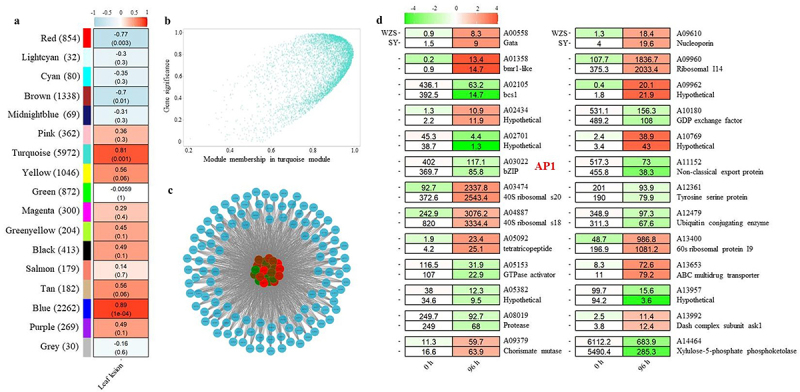
WGCNA to identify hub genes during infection.

To date, the infection mechanism of *C. fructicola* on *C. oleifera* is not fully understood. Thus, we primarily use RNA-seq data from different geographical populations of *C. fructicola* to investigate the hub genes correlated with leaf lesion size [24]. Notably, the pathogenicity of *C. fructicola* from Wuzhishan (WZH) has a more significant impact on oil tea leaf tissue compared to the population from Shaoyang (SY) [24]. We filtered out 14,437 genes with small fluctuations using the “goodSamplesGenes” function and identified 17 separate modules of related genes distinguished by different colours (Figure 1a). The gene counts of the 17 modules ranged from 30 to 5,972, with the largest module being “turquoise,” which was highly correlated with leaf lesion size (0.81). The association between Module Membership (turquoise) and Gene-Trait correlation was plotted in Figure 1b. Using the cluster algorithm in WGCNA, we identified 33 genes that highly connect the intra-module genes in module turquoise (Figure 1c).

a. WGCNA module-phenotypes correlation analysis b. Module membership in turquoise module. c. Networks of hub genes in module turquoise, d. Heatmap of hub genes in modules during infection of *C. fructicola* on oil-tea leaves. The heatmap represents the expression pattern of genes in response to cold stress. Relative expression levels were calculated as a Log2-fold change against CK (see Section Materials and Methods). The red colour shows an upregulation of a given gene, and the green indicates a downregulation. Labeled number in each tile is the expression level “FPKM.”

“The pattern of expression of these genes during infection varies, with some being up-regulated and others down-regulated, despite being part of the same co-expression module. Additionally, these genes are predicted to have diverse biological functions, contributing to various aspects of the plant’s response to infection (Figure 1d). Finally, we selected the predicted bZIP transcription factor gene A03022 for further characterisation to investigate the regulatory network. The *CfAP1*(A03022) gene is 1804 base pairs long and encodes a protein of 566 amino acids. The protein consists of one basic leucine zipper domain (BRLZ) and two PAP1 domains, as shown in Figure S1a. Phylogenetic analysis indicates that *CfAP1* is closely clustered with another bZIP transcription factor, AP1, in *C. gloeosporioides* (as shown in Figure S1b).

### CfAp1 plays a pivotal role in C. fructicola’s growth, stress response, and pathogenicity

**Figure 2.**
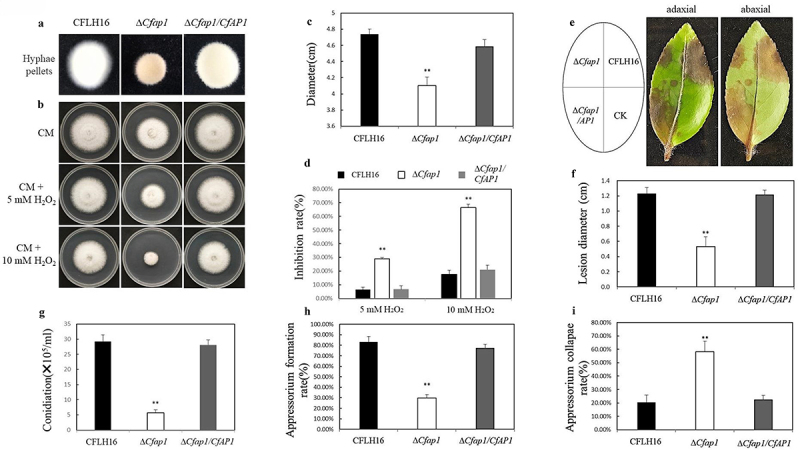
The loss of *CfAP1* led to the weakening of the pathogenicity of *C. fruitiflora*.

We examine the impact of CfAp1 on a range of physiological and pathogenic traits in our effort to define the function, with an emphasis on pathogenicity, oxidative stress response, vegetative growth, and asexual reproduction (Figure 2). Comparing the Δ*Cfap1* mutant with the wildtype strain CFLH16 and the complemented strain Δ*Cfap1*/*AP1* on CM, we found that the Δ*Cfap1* mutant diminished aerial hyphae (Figure 2a) and demonstrated significantly slower growth (Figure 2b,c). This references to growth abnormalities brought on by *CfAP1* deletion. Thus, the absence of *CfAP1* markedly hampers the spore-producing ability of Δ*Cfap1*. *AP1* has been shown to respond to H_2_O_2_ stress and regulate intracellular ROS homoeostasis in both yeast and *M. oryzae*. We speculate that this mechanism may also be present in *C. fructicola* [25–27]. Next, we explored the role of *CfAP1* in handling oxidative stress, a critical aspect of pathogenicity during plant invasion [28–30]. The H_2_O_2_ treatment revealed that the Δ*Cfap1* mutant displayed significantly reduced tolerance to oxidative stress. Mycelial growth was notably inhibited on CM plates with 5 mm and 10 mm H_2_O_2_ (Figure 2b), with inhibition rates increasing by 10.9% (5 mm) and 37.5% (10 mm) compared to CFLH16 and the complemented strains (Figure 2d).

a. The hyphae of the mutant strain were sparse and loose, while those of the wildtype strain were tightly intertwined. b. The growth of the wildtype strain (CFLH16), the Δ*Cfap1* mutant strain, and the Δ*Cfap1*/*CfAP1* complemented strain on CM with 0/5/10 mm H_2_O_2_ at 28°C in the dark for 2 days. c. Statistical analysis of the difference in colony diameter among the CFLH16, the Δ*Cfap1* mutant strain, and the Δ*Cfap1*/*CfAP1* complemented strain on CM (**: *p* < 0.01). d. Statistical analysis of the growth inhibition rate of the strains under H_2_O_2_ stress (**: *p* < 0.01). e. The lesions caused by the CFLH16, the Δ*Cfap1* mutant strain, and the Δ*Cfap1*/*CfAP1*complemented strain on unwound tea leaves. f. Statistical analysis of the difference in lesion diameter (**: *p* < 0.01). g. Statistical analysis of the conidiation (2 days) of CFLH16, the Δ*Cfap1* mutant strain, and the Δ*Cfap1*/*CfAP1* complemented strain. h. Statistical analysis of the appressorium formation rate (24 h) of the CFLH16, the Δ*Cfap1* mutant strain, and the Δ*Cfap1*/*CfAP1* complemented strain. Statistical analysis of the rate of appressorium formation on hydrophobic slides. I. Statistical analysis of the rate of appressorium formation collapse (**: *p* < 0.01).

In terms of pathogenicity, when the CFLH16, Δ*Cfap1* mutants, and complemented strains of *C. fructicola* were inoculated onto unwounded leaves, the Δ*Cfap1* mutant was found to cause significantly smaller lesions, averaging 0.53 cm in diameter on fresh tea-oil leaves, after three days (Figure 2e, f). We assessed the conidiation of the Δ*Cfap1* mutant and found a considerable decrease in conidia formation relative to the CFLH16 and Δ*Cfap1*/*AP1*, since asexuality is a crucial component of the anthracnose cycle (Figure 2g). Finally, we delved into the role of CfAp1 in appressorium formation and expansion pressure, critical for plant invasion. The Δ*Cfap1* mutant formed a substantially lower number of appressoria, only 38.4% of the wildtype CFLH16 count (Figure 2h, *p* ≤ 0.01). A greater collapse rate of appressoria under the impact of 2 mol/L Glycerol was observed in Δ*Cfap1* (Figure 2i), suggesting that the reduction in pathogenicity may be associated with decreased appressorium expansion pressure. In summary, these findings underscore the pivotal role of CfAp1 in vegetative growth, asexual reproduction, oxidative stress response, pathogenicity, as well as appressorium formation and pressure in *C. fructicola*.

### Rna-seq analysis of ΔCfap1 under H_2_O_2_ treatment

**Figure 3.**
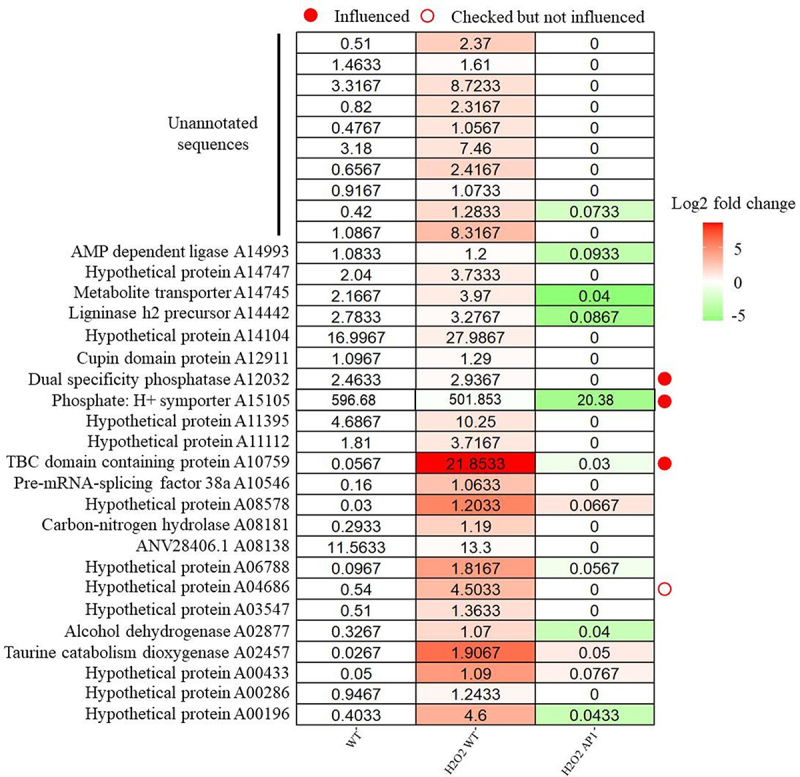
Candidate genes selected from AP1 H_2_O_2_ treatment rna-seq data.

To gain a detailed understanding of the transcriptome changes in Δ*Cfap1*, as well as to identify the functional genes involved in the oxidative stress response in *C. fructicola*, Δ*Cfap1* and wildtype were treated with 2.5 mm H_2_O_2_ for 1 day. Obviously, most redox genes in Δ*Cfap1* are differentially expressed after treatment, whereas genes during infection have the opposite tendency (Figure S2 and S3). With a threshold of FPKM > 1 in WT (H_2_O_2_ treated) and FPKM < 0.1 in Δ*Cfap1* (H_2_O_2_ treated), we identified 33 genes may under regulation of *CfAP1*. Real-time fluorescence quantification showed that A10759, A15105 and A12032 expression decreased significantly in the mutant Δ*Cfap1* under 2.5 mm H_2_O_2_ stress, consistent with transcriptome (Figure 3 and Figure S4b). Pathogenicity test shows that A10759, A15105 and A12032 exhibited weaken pathogenicity (Figure S4d) and only A12032 differed in oxidative stress response. This is supported by the observation that the “turquoise” module, which is positively correlated with leaf lesion size with a correlation coefficient of 0.8 (Figure 1a), primarily functions in “oxidoreductase activity” within the “nucleus,” as revealed by GO enrichment analysis (Figure S5).

The heatmap represents the expression pattern of genes in response to cold stress. Relative expression levels were calculated as a Log2-fold change against CK (see Section Materials and Methods). The red colour shows an upregulation of a given gene, and the green indicates a downregulation. Labeled number in each tile is the expression level “FPKM.”

### Identification of CfMsg5 and phylogenetic tree analysis; subcellular localization of CfAp1 and CfMsg5

**Figure 4.**
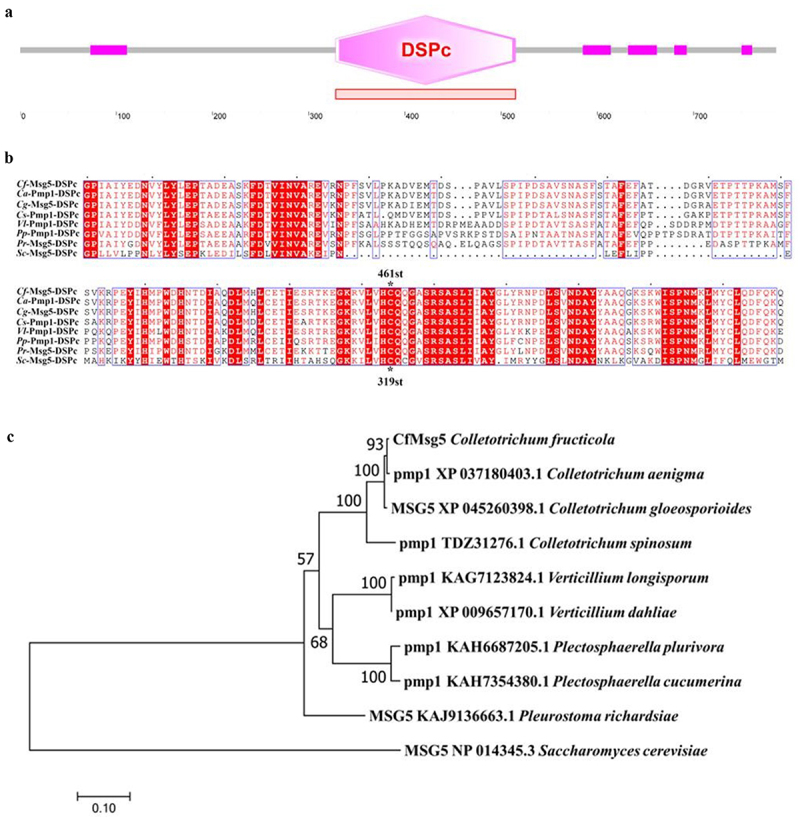
CfMsg5 capital domain and phylogenetic analysis.

The *CfMSG5* gene is 2361 bp in length and encodes a protein comprising 786 amino acids. This protein contains one Dual specificity phosphatase, catalytic domain (DSPc), and five domains of unknown function (Figure 4a). Comparative analysis of the DSPc sequence of several species revealed that, the active site HC motif (Ile/Val-His-CysXXXXXArg) containing protein tyrosine phosphatase (PTPases) [31,32] is highly conserved in fungi, And the cysteine is located at 461st amino acid of CfMsg5 (Figure 4b). Phylogenetic analysis revealed a high amino acid sequence identity with *C. viniferum* and a lesser yet significant sequence identity with *S. cerevisiae* Msg5 (Figure 4c), suggesting conservation of Msg5 proteins across fungal species.

a. The CfMsg5 capital domain was found using the SMART website (http://smart.embl-heidelberg.de/); DSPc: Dual specificity phosphatase, catalytic domain; the number denotes the amino acid’s serial number, pink boxes represent an unknown domain. b. Amino acid sequence alignment of DSPc domain of *Colletotrichum fructicola* Msg5, *Colletotrichum* aenigma pmp1, *Colletotrichum* gloeosporioides Msg5, *Colletotrichum* spinosum Pmp1, *Verticillium longisporum* Pmp1, *Plectosphaerella plurivora* Pmp1, *Plectosphaerella cucumerina* Msg5 and *Saccharomyces cerevisiae* Msg5. Red background indicates the residues that are completely identical. The conserved amino acid residues are boxed. Dashes are inserted to allow for maximal alignment of the sequences. Asterisks and numbers represent mutation sites. c. The phylogenetic tree was built using the neighbour-joining (NJ) technique and analysed in Mega7.0 with 1,000 bootstrap repetitions; the number on the branch represents the support determined with 100 bootstrap replicates in the NJ analysis; the scale bar represents the branch length. The number in front of each reference taxon is the GenBank accession number of the orthologous amino acid sequence to CfMsg5.

**Figure 5.**
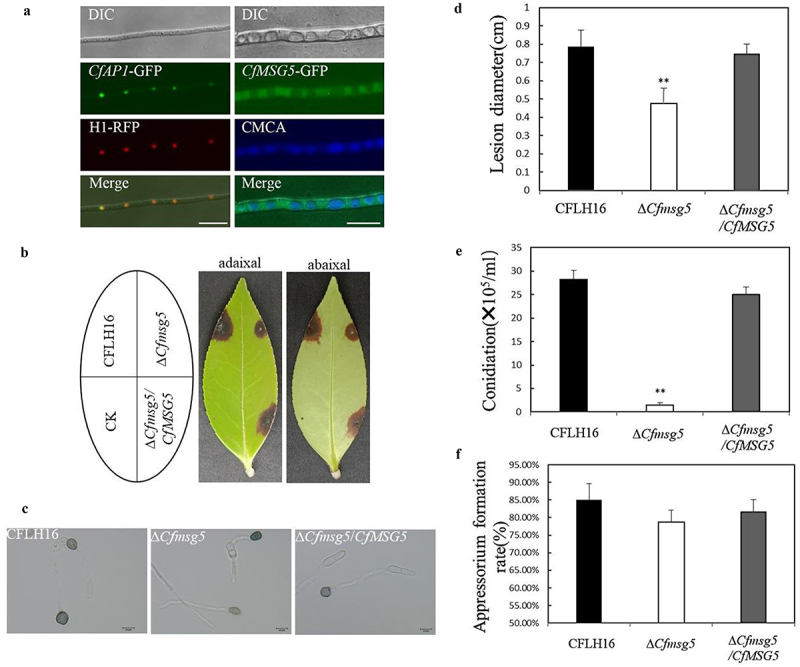
The pathogenicity of *C. fructicola* was regulated by CfMsg5.

To obtain a comprehensive understanding of the intracellular distribution patterns of the encoded proteins and their potential interactions within host cells, we have designed subcellular localization experiments that aim to directly visualize the spatial localization of CfAp1 and CfMsg5 proteins within the cellular environment. Our results revealed that CfAp1-GFP colocalized with H1-RFP, a nucleus marker, in the hyphae under nutrient-rich CM conditions (Figure 5a). However, in the complementation strain Δ*Cfmsg5*/*CfMSG5*, the green fuorescence was evenly distributed in the cytoplasm of mature hyphae and the CfMsg5-GFP was not localized to the vacuole membrane of hyphae (Figure 5a). These results indicates that CfAp1 is localized in the nucleus, while CfMsg5 is distributed in the cytoplasm excluding vacuoles. The localization of CfAp1 in the nucleus suggests its involvement in gene expression regulation. Meanwhile, the presence of CfMsg5 in the cytoplasm suggests that it may perform a range of functions such as signal transduction.

a. H1 was used as a nucleus marker. The colocalization of CfAp1-GFP and H1-RFP showed that CfAp1 localized to the nucleus in hyphae. Hyphae of ∆*Cfmsg5*/*CfMSG5* strain were incubated on liquid medium for 24 h.7-Amino-4-chloromethylcoumarin (CMAC) staining of vacuole was performed at the 37°C for 30 min. CfMsg5 is distributed evenly throughout the cytoplasm of mature hyphae without localization to the vacuole membrane. Bar = 10 um. b. The lesions caused by the wildtype strain (CFLH16), the Δ*Cfmsg5* mutant strain, and the complemented strain on wounded tea-oil leaves. c. The appressorium of the wildtype strain (CFLH16), the Δ*Cfmsg5* mutant strain, and the ∆*Cfmsg5*/*CfMSG5* complemented strain; Bar = 10 μm. d. Significant difference in lesion diameter based on statistical analysis (**: *p* < 0.01). e. Statistical analysis of the conidiation (2 days) of the wildtype strain (CFLH16), the Δ*Cfmsg5* mutant strain, and the ∆*Cfmsg5*/*CfMSG5* complemented strain (**: *p* < 0.01). f. Statistical analysis of appressorium formation rate (24 h) of the wildtype strain (CFLH16), the Δ*Cfmsg5* mutant strain, and the ∆*Cfmsg5*/*CfMSG5* complemented strain (*p* > 0.05). Error bars, SD. Data presented are the mean from three independent biological experiments.

### CfMsg5 plays an important role in C. fructicola pathogenicity, ROS clearance, and CWI MAPK cascade regulation

The significance of CfMsg5 in the pathogenicity of *C. fructicola* was established when it was observed that the Δ*Cfmsg5* mutant strain resulted in smaller lesions (average diameter of 0.48 cm) on tea-oil leaves in comparison to the CFLH16 and complementation strain Δ*Cfmsg5*/*CfMSG5*, indicating a reduction in pathogenicity (Figure 5b,d). The conidiation rate in the Δ*Cfmsg5* mutant was reduced by approximately 95% compared to the CFLH16 and complementation strain Δ*Cfmsg5*/*CfMSG5*, indicating that the absence of CfMsg5 severely inhibits spore production (Figure 5e). However, CfMsg5 was not discovered to have an influence on appressorium production since the rate of formation in mutant Δ*Cfmsg5* was 78.67% (Figure 5c,f), similar to that in the CFLH16 and complementation strain Δ*Cfmsg5*/*CfMSG5*.

**Figure 6.**
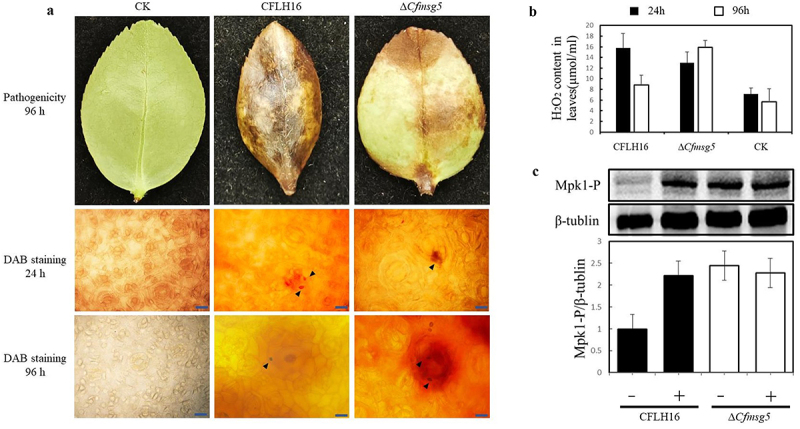
CfMsg5 plays an important role in *C. fructicola* pathogenicity, ROS clearance, and CWI MAPK cascade regulation.

Thus, we assumed that the reduced pathogenicity of Δ*Cfmsg5* mutants is caused by other causes. Using conidia to infect leaves, hydrogen peroxide content in leaves was determined and DAB stained was observed. DAB combines with H_2_O_2_ to form a reddish-brown precipitate. And after 24 hours, the content of ROS in both Δ*Cfmsg5* mutant and wild-type infested leaves was significantly increased compared with that of uninfected leaves (Figure 6b). Moreover, there was no significant difference in the content of reddish-brown precipitate and ROS in mutant and wild-type infested leaves (Figure 6a,b), indicating that the degree of PTI caused by wild-type and mutant was similar in the initial stage of infection. After 96 hours, it was observed that the mutant strain exhibited elevated levels of ROS at the infection points. The higher levels of reddish-brown precipitate in mutants compared to wildtype or healthy leaves indicated a more substantial accumulation of H_2_O_2_ in the mutants (Figure 6a). This is also confirmed by the determination of H_2_O_2_ content in leaves (Figure 6b). These findings suggested that the CfMsg5 protein is necessary for effective ROS clearance during the infection process.

a. Conidial suspension (10^6^ conidia/ml) of CFLH16 and Δ*Cfmsg5* were sprayed on tea-oil leaves. CK: sterile water was sprayed on tea-oil leaves as a control. Diseased leaves were harvested 96 hours after inoculation; DAB staining after 24 hours and 96 hours. The black arrow is appressorium. Bar = 25 μm. b. H_2_O_2_ content were determined using visible spectrophotometry in leaves. Wavelength: 415 nm. c. The phosphorylation Level of Mpk1was determined using western blotting, before (-) and 30 min after addition of 5 mm H_2_O_2_ (+), using immunoblot analysis with antiphospho-p44/42 MAPK antibody (p-Mpk1). β-tubulin antibody was used as loading control. Ratio of pMpk1 versus β-tubulin levels, normalized to the WT strain at time 0. Error bars, SE. Data presented are the mean from three independent biological experiments.

Under conditions of hydrogen peroxide stress, we tested the role of CfMsg5 in regulating Mpk1 phosphorylation in *C. fructicola*. Western blot analysis with a phosphor specific antibody detected an approximately two-fold increase in Mpk1 phosphorylation levels in CFLH16 (Figure 6c). And it was found that the phosphorylation levels of the MAPK Mpk1 were constitutively elevated in the Δ*Cfmsg5* mutant, even in the absence of H_2_O_2_ treatment. These elevated levels did not change further after the addition of hydrogen peroxide, implying that the CfMsg5 protein negatively regulates the CWI MAPK pathway in the absense of oxidative stress, and stimulation of hydrogen peroxide causes relieve CfMsg5’s inhibitory effect on the CWI pathway (Figure 6c). Subsequently, the study demonstrated the crucial role of CfMsg5 in the conidiation process of *C. fructicola*. In summary, these findings demonstrate that CfMsg5 plays an important role in pathogenicity of *C. fructicola*, ROS clearance, conidiation, and the regulation of the CWI MAPK cascade but does not significantly influence the formation of the appressorium.

### CfMsg5 regulates optimal growth, various stress response and unfolded protein response.

**Figure 7.**
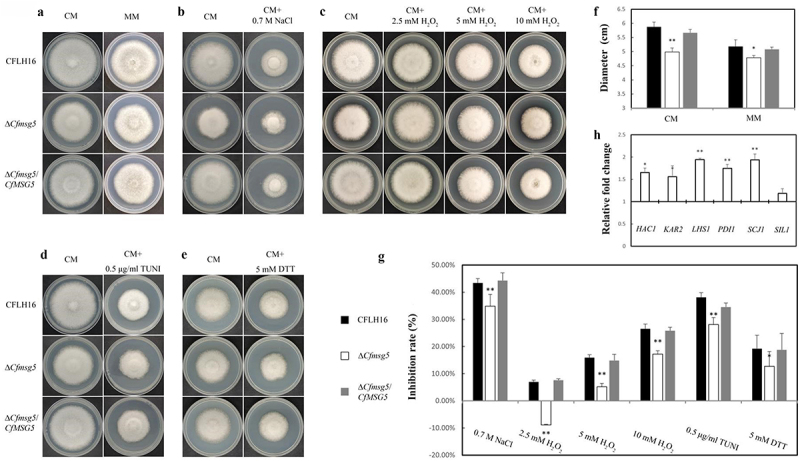
CfMsg5 regulates optimal growth, various stress response and unfolded protein response.

The significance of CfMsg5 in growth of *C. fructicola* was demonstrated when the Δ*Cfmsg5* mutant showed slower colony growth than both the CFLH16 and Δ*Cfmsg5*/*CfMSG5* on complete medium (CM) and minimal medium (MM) (Figure 7a,f). To further elucidate the role of CfMsg5, the inhibition rate of the ∆*Cfmsg5* mutant was measured under various stress conditions, including osmotic stress (0.7 M NaCl; inhibition rate: 34.8%), oxidative stress (2.5 mm, 5 mm and 10 mm H_2_O_2_; inhibition rate: −8.9%, 5.2% and 17.2%), and endoplasmic reticulum (ER) stress (0.5 μg/ml Tunicamycin and 5 mm DTT; inhibition rate: 28.1% and 12.7%). Surprisingly, inhibition rate of these stresses on mutants ∆*Cfmsg5* was significantly lower than that of CFLH16 and ∆*Cfmsg5*/*CfMSG5*, even the growth of ∆*Cfmsg5* was promoted in 2.5 mm H_2_O_2_ treatment (Figure 7b-e, and 6g). These indicated that CfMsg5 is negatively regulates the response of *C. fructicola* to osmotic stress, oxidative stress, ER stress. The study also investigated the unfolded protein response (UPR) in *C. fructicola*, a cellular stress response related to ER stress. The expression levels of several genes involved in the UPR were assessed using qPCR. While the mRNA levels of *SIL1*(an ER protein) remained unchanged, the expression levels of *HAC1* (a transcription factor gene for UPR), *KAR2*(an ER molecular chaperone), *PDI1* (protein disulphide isomerase 1), *SCJ1* (chaperone dnaJ 1), and *LHS1* (an ER molecular chaperone) were significantly upregulated in the ∆*Cfmsg5* mutant (Figure 7h). The results suggest that CfMsg5 May negatively regulate the transcription factor *CfHAC1* to act to negatively regulate the UPR. In summary, these findings suggested that CfMsg5 plays a crucial role in optimal growth various stress response, and unfolded protein response.

a: The growth rate of ∆*Cfmsg5* was significantly reduced in both CM and MM media in the dark for 3 days. b. Growth status of CFLH16, ∆*Cfmsg5* and ∆*Cfmsg5*/*CfMSG5* complemented strain in the dark for 3 days under 0.7 M NaCl stress. c: Growth status of CFLH16, ∆*Cfmsg5* and ∆*Cfmsg5*/*CfMSG5* complemented strain on CM with 0/2.5/5/10 mm H_2_O_2_ at 28°C in the dark for 3 days. d: Growth status of wildtype strain (CFLH16), ∆*Cfmsg5* mutant, and ∆*Cfmsg5*/*CfMSG5* complemented strains on CM supplemented with 0.5 μg/ml TUNI at 28°C in the dark for 3 days. e: Growth status of wildtype strain (CFLH16), ∆*Cfmsg5* mutant, and ∆*Cfmsg5*/*CfMSG5* complemented strains on CM supplemented with 5 mm DTT at 28°C in the dark for 2 days. f: Statistical analysis of diameter of strains colony in CM and MM (**: *p* < 0.01; *: 0.05<*p* < 0.01). g: Statistical analysis of growth inhibition rate of strains in different stress (**: *p* < 0.01; *: 0.05<*p* < 0.01). h: Expression level analysis of 6 selected genes in *C. fructicola*. Significant differences compared with the wildtype were estimated (**: *p* < 0.01). Error bars, SD. Data presented are the mean from three independent biological experiments.

### The dephosphorylation site affects the dephosphorylation of Mpk1 by CfMsg5, which in turn affects the pathogenicity and response to stress of C. fructicola

**Figure 8.**
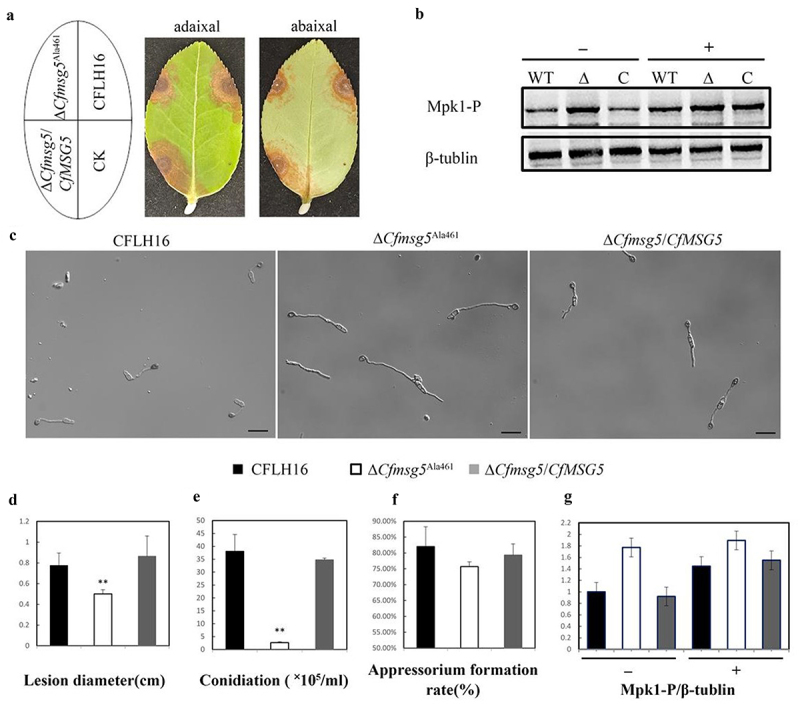
Phosphatase activity of CfMsg5 is significant to pathogenicity and CWI MAPK cascade regulation.

Cysteine residues in the HC motif are absolutely necessary for phosphatase activity [33,34]. In *S. cerevisiae*, Doi performed site-directed in vitro mutagenesis to change the Msg5 codon for Cys319 to Ala319 [31], that inactivates dual specificity phosphatase Msg5. And the analysis of amino acid sequences showed that CfMsg5 also contained a sequence related to the HC motif (Ile/Val-His-CysXXXXXArg). Then we performed site-directed mutagenesis to change the CfMsg5 for Cys461 to Ala461. It was found that the lesion size of the point mutant ∆*Cfmsg5*^Ala461^ was about 0.45 cm on the tea-oil leaves with wound, which was significantly smaller than that of CFLH16 and ∆*Cfmsg5*/*CfMSG5* (Figure 8a,d). These results indicated that the phosphatase activity of CfMsg5 affected pathogenicity of *C. fructicola*. ∆*Cfmsg5*^Ala461^ had a conidia concentration of approximately 2.67×10^5^ conidia/ml after 2 days of shake flask culture (Figure 8e). ∆*Cfmsg5*^Ala461^ was reduced by approximately 92% compared to the CFLH16 and complementation strain Δ*Cfmsg5*/*CfMSG5*, indicating that phosphatase inactivation of CfMsg5 severely reduced the production of conidia. However, the phosphatase inactivation of CfMsg5 was not discovered to have an influence on appressorium production since the rate of formation in mutant ∆*Cfmsg5*^Ala461^ was 75.67% (Figure 8c,f), similar to that in the CFLH16 and complementation strain Δ*Cfmsg5*/*CfMSG5*. These results are the same as those of the gene deletion mutant ∆*Cfmsg5*.

a. The lesions caused by the CFLH16, the Δ*Cfmsg5* ^Ala461^ mutant strain, and the ∆*Cfmsg5*/*CfMSG5* complemented strain on wounded tea-oil leaves. b and g: The phosphorylation Level of Mpk1 was determined using western blotting, before (-) and 30 min after addition of 5 mm H_2_O_2_ (+), using immunoblot analysis with antiphospho-p44/42 MAPK antibody (p-Mpk1). β-tubulin antibody was used as loading control. Ratio of pMpk1 versus β-tubulin levels, normalized to the WT strain at time 0; Error bars, SE (WT:CFLH16;Δ:Δ*Cfmsg5* ^Ala461^ mutant; c: the ∆*Cfmsg5*/*CfMSG5* complemented strain). c: The appressorium of the CFLH16, the Δ*Cfmsg5* ^Ala461^ mutant strain, and the ∆*Cfmsg5*/*CfMSG5* complemented strain; Bar = 20 μm. d: Significant difference in lesion diameter based on statistical analysis (**: *p* < 0.01). e: Statistical analysis of the conidiation (2 days) of the CFLH16, the Δ*Cfmsg5* ^Ala461^ mutant strain, and the ∆*Cfmsg5*/*CfMSG5* (**: *p* < 0.01). f: Statistical analysis of appressorium formation rate (24 h) of the CFLH16, the Δ*Cfmsg5* ^Ala461^ mutant strain, and the ∆*Cfmsg5*/*CfMSG5* (*p* > 0.05). Error bars, SD. Data presented are the mean from three independent biological experiments.

Western blot analysis showed that the phosphorylation level of Mpk1 in ∆*Cfmsg5*^Ala461^ was increased compared with CFLH16 and ∆*Cfmsg5*/*CfMSG5* (Figure 8b,g). It was consistent with that of the mutant ∆*Cfmsg5*. Although the phosphorylation level of Mpk1 in the mutant ∆*Cfmsg5*^Ala461^ did not change significantly under the stress of hydrogen peroxide, the phosphorylation level of Mpk1 in CFLH16 and ∆*Cfmsg5*/*CfMSG5* was significantly increased (Figure 8b,g). These indicated that CfMsg5 dephosphorylated Mpk1 in the absence of hydrogen peroxide stress, negatively regulating the CWI MAPK pathway. Stimulated by hydrogen peroxide, the CWI MAPK pathway is activated.

**Figure 9.**
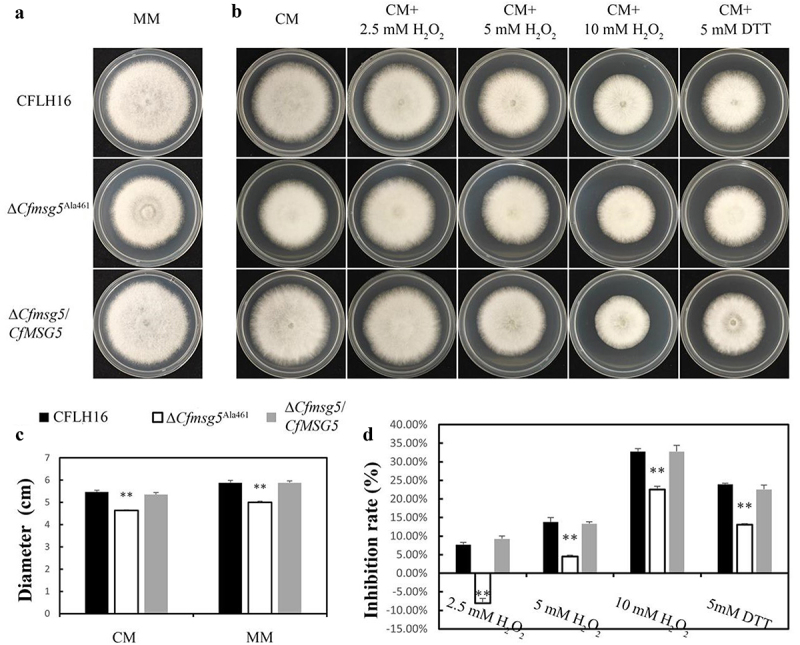
Phosphatase activity of CfMsg5 is important to stress response.

To prove that the mutant ∆*Cfmsg5* growth slows down because of phosphatase activity, we inoculated the strains to CM and MM. The ∆*Cfmsg5*^Ala461^ mutant showed slower colony growth than both CFLH16 and ∆*Cfmsg5*/*CfMSG5* on CM and MM (Figure 9a-c). The inhibition rate of the ∆*Cfmsg5* mutant was measured under various stress conditions, including oxidative stress (2.5 mm, 5 mm and 10 mm H_2_O_2_; inhibition rate: −8.1%, 4.5% and 22.6%), and endoplasmic reticulum (ER) stress (5 mm DTT; inhibition rate: 13.1%) (Figure 9d). Inhibition rate of these stresses on mutants ∆*Cfmsg5* ^Ala461^ was significantly lower than that of CFLH16 and ∆*Cfmsg5*/*CfMSG5*, even the growth of ∆*Cfmsg5* ^Ala461^ was promoted under 2.5 mm H_2_O_2_ treatment (Figure 9b,d). These results are the same as those of the gene deletion mutant ∆*Cfmsg5*. These indicated that the dephosphorylation site of CfMsg5 play a significant role in the response of *C. fructicola* to oxidative stress and ER stress.

a: The growth rate of ∆*Cfmsg5* ^Ala461^ was significantly reduced in MM media in the dark for 4 days. b. Growth status of CFLH16, ∆*Cfmsg5* and ∆*Cfmsg5*/*CfMSG5* complemented strain in the dark for 3 days under 0/2.5/5/10 mm H_2_O_2_ stress or 5 mm DTT. c: Statistical analysis of diameter of strains colony in CM and MM (**: *p* < 0.01). d: Statistical analysis of growth inhibition rate of strains in different stress (**: *p* < 0.01). Error bars, SD. Data presented are the mean from three independent biological experiments.

## Discussion

Pathogen invasion is effective when pathogen-associated molecular patterns (PAMPs) are translocated to hosts, suppressing the plant’s immune system and enabling the pathogen to penetrate and establish itself [15,35–37]. In contrast, plants activate pattern recognition receptors (PRRs) and immune receptors (NLRs) to recognize these PAMPs, subsequently activating hypersensitivity reactions (HR), reactive oxygen species (ROS) bursts, and MAPK signalling pathways. During plant–pathogen interactions, ROS generation around the infection site is one of the early hallmarks of pattern-triggered immunity (PTI). A significant ROS burst plays a dual role: it induces cell wall strengthening and enhances antimicrobial activity, and acts as a diffusible secondary signal to induce various plant defence responses. These responses include the accumulation of pathogenesis-related (PR) proteins in surrounding cells, the synthesis of phyto-defensins, and the broadening of programmed cell death (PCD) [38]. Localized PCD in infected cells, triggered by H_2_O_2_, limits fungal spread [39,40]. The H_2_O_2_ signal pathway triggers the MAPK cascade, followed by downstream transcriptome responses. To counteract this oxidative stress, pathogens have evolved anti-oxidation systems. Among these, the bZIP transcription factor Ap1 (Activating protein 1) in pathogenic fungi responds to oxidative stresses and regulates multiple antioxidant genes to clear ROS. Ap1’s involvement in ROS clearance significantly impacts pathogenicity in multiple pathogens [16,41]. Therefore, understanding the mechanism of AP1 (CfAp1)-mediated ROS elimination is crucial for an in-depth understanding of the pathogenic mechanism of *C. fructicola* in anthracnose.

We used WGCNA to explore the gene association pattern between transcriptome data of two oil-tea cultivars with their infected anthracnose leaves lesion region in order to identify the hub module(s)/genes involved in *C. fructicola* pathogenesis and resistance to host ROS burst. In Figure 1, we observed that four ribosomal proteins, A03474, A04887, A09960, and A13400, were significantly and highly up-regulated during plant–pathogen interactions, indicating a high activity of ribosome biogenesis and protein synthesis, which is the fundamental rate-limiting step for fungi growth and proliferation [42]. Other genes that encode proteins may contribute in the stimulation of metabolite processes include GTPase activator, protease, and xylulose-5-phosphate phosphoketolase. Interestingly, only two transcription factors, A00558 (GATA) and A03022 (bZIP), were identified as hub genes in module turquoise (5972 genes, the largest module) (Figure 1d), in which A03022 was identified as an AP1 homolog. As member of bZIP transcription factor, AP1 was characterized as the major oxidative stress regulator and one of the immediate targets for regulation by SAPK/MAPK in yeast and filamentous fungi [43–46]. Thus, we hypothesized that CfAp1 plays a regulatory role in oxidative stress response, because of “turquoise” module are positive correlated leaves lesion region with 0.8 (Figure 1a), and WGCNA result shows that GO enrichment analysis predicts that the Turquoise module is enriched in genes encoding proteins that localize to the nucleus and exhibit oxidoreductase activity. (Figure S3).

In this study, we examined CfAp1, a key regulator in oxidative stress responses. Our analysis under H_2_O_2_ treatment revealed nuanced expression patterns of redox-related genes in the Δ*Cfap1* strain. While Figure S4 shows an opposite expression pattern for some genes compared to the wild-type, Figure S5 indicates a more complex scenario where approximately half of the redox-related genes exhibit similar expression patterns in both strains. This suggests that the deletion of CfAp1 does not uniformly reverse gene expression but rather results in a mixed response, with some genes showing opposite expression patterns and others maintaining similarity. This complexity underscores the intricate role of CfAp1 in modulating the oxidative stress response and its impact on pathogenicity in the host oil-tea (Figure 2b,d).

These observations collectively emphasize the conserved role of AP1 in the early stages of infection across diverse pathogenic fungi. Genes that are up-regulated during infection, but down-regulated in the Δ*Cfap1* mutant under H_2_O_2_, could contribute to *C. fructicola*‘s resistance to ROS. Our study further identified genes A12032, A10759, and A15105 as significantly reduced or even absent in Δ*Cfap1*. Out of these, three quarters appear to influence pathogenicity, while one gene is related to H_2_O_2_ resistance. In silico analysis showed A12032 as a homolog of the dual specificity phosphatase (DSP) gene *MSG5* from *Saccharomyces cerevisiae* (Figure 4a,b). We previously described how external stimuli like ROS amplify protein phosphorylation signals via the MAPK signaling pathway, where CfMsg5 serves as a limiting factor. Various physical and chemical stressors activate the Cell Wall Integrity (CWI) pathway, triggering Slt2-specific MAPKKs – the substrate for Msg5. Interestingly, yeast was the first to identify Msg5 as a DSP that aids in pheromone response recovery by deactivating Fus3 [31]. The subsequent discovery of Ptp2 and Ptp3 as negative regulators of Fus3 marked the first instance of a MAPK regulated by both PTPs and DSPs [47]. Furthermore, Msg5’s role in pheromone adaptation was broadened by the revelation of its contribution to maintaining low basal phosphorylation in Fus3 [48]. When overexpressed, Msg5 can decrease Kss1 phosphorylation. It also governs signalling via the cell integrity pathway by acting on the MAPK Slt2 [49,50]. Under standard conditions, Msg5 appears to be the most effective Slt2 phosphatase. It does not seem to influence Hog1 [51]. These findings position Msg5 as a comprehensive negative regulator of yeast MAPKs.

AP1 is recognized for binding to relatively conserved cis-regulatory elements, yet our investigation revealed no evidence of AP-1 binding to cis-elements in the *CfMSG5* promoter region. Furthermore, it is noteworthy that the distinct localization patterns of CfAp1 and CfMsg5 within the cell imply that they may function independently or, in some instances, potentially collaborate or regulate each other’s functions. This collaborative relationship may involve their participation in a shared cellular process or the regulation of each other’s functions through interactions. We also observed the absence of redundancy and alternative pathways responding to ROS, as the *MSG5* gene failed to express in *AP1* mutant strains. This leads us to propose that a potential Msg5-MAPK-AP1 module may play a pivotal role in the response to ROS. This discovery gives rise to questions surrounding the existence of a new regulatory network in *C. fructicola* that responds to ROS, warranting further research. Elucidating the exact molecular interactions between CfAp1 and CfMsg5 could provide deeper insights into how *C. fructicola* manages oxidative stress during infection. Given that CfAp1 is localized in the nucleus and CfMsg5 in the cytoplasm, understanding how these two proteins might communicate or regulate each other’s activity despite their spatial separation is a key area for future research. Investigating whether CfMsg5 modulates MAPK pathway activity indirectly through other signalling intermediaries or whether it has a direct effect on nuclear targets could reveal novel aspects of fungal pathogenicity. Furthermore, exploring the potential feedback mechanisms within this regulatory module could offer new strategies for disrupting the oxidative stress response in *C. fructicola*, ultimately leading to novel antifungal targets. Collectively, our findings offer insights into the molecular mechanisms underpinning ROS response in *C. fructicola*, which could potentially extend to other species. After mutating the *CfMSG5* gene, we noticed a considerable decline in pathogenicity compared to wild-type and complemented strains (Figure 5b,d). However, the formation of appressorium in the ∆*Cfmsg5* mutant remained unaffected (Figure 5f). In contrast, the ∆*Cfmsg5* mutants’ capacity to scavenge ROS at the infection sites in the host was substantially reduced. This suggests that external stresses, particularly ROS, are primarily responsible for the reduced pathogenicity of the ∆*Cfmsg5* mutant.

The endoplasmic reticulum (ER), crucial for protein folding and processing, is fundamental for fungal development and pathogenicity. In *C. fructicola*, ∆*Cfmsg5* exhibited markedly reduced hyphal growth, decreased conidiation, and weakened virulence (Figure 6a,b,e,f). Tunicamycin (TUNI) and DTT, two ER stress inducers that alter protein folding through different mechanisms, were less impactful on the ∆*Cfmsg5* strain. The reasons behind the growth recovery of ∆*Cfmsg5* under DTT and TUNI treatment remain ambiguous, necessitating further investigation. In *P. oryzae*, the ER molecular chaperone PoLHS1-deletion mutant exhibited deficiencies in sporulation, appressorium-mediated penetration, and biotrophic invasion [52]. However, *LHS1* was notably upregulated in ∆*Cfmsg5*, as were *SCJ1* and *PDI1*, two UPR components. Indication of UPR activation in organisms is signified by up-regulation of the marker protein, Kar2/Bip [53]. The expression level of *KAR2*/BIP significantly increased in ∆*Cfmsg5* while it decreased in ∆*Pohac1* [54]. The expression level of *HAC1* also significantly increased in ∆*Cfmsg5* (Figure 6g). Intriguingly, proteins misfolded in the ER are degraded by UPR, which augments protein-folding reactions through the transcriptional up-regulation of genes encoding ER-associated degradation (ERAD) components [55,56], or in a UPR-independent manner in *S. cerevisiae* [57]. In summary, it seems that CfMsg5 May restrain the UPR pathway.

Protein disulphide isomerase (PDI), a catalyst for native disulphide bond formation in the ER lumen, has been observed to participate in dithiol-disulphide exchange reactions catalysing dithiol oxidation, disulphide reduction, or disulphide isomerization depending on the nature of the substrate protein and the redox conditions of the assay [58]. In *S. cerevisiae*, Pdi1 acts as an oxidase in vivo [59]. In ∆*Cfmsg5* mutants, *PDI1* was significantly up-regulated. Consequently, we hypothesize that disulphide bond formation may relate to ROS signalling and that CfMsg5 might inhibit ROS conduction, although this theory still requires validation. UPR defective mutants in yeast have been associated with defects in cell wall integrity [60]. Mitogen-activated protein kinase (MAPK) cascades are key signalling modules controlling development and pathogenicity in fungal pathogens [61]. The CWI MAPK, Mpk1, is essential for sensing plant root exudates as well as for host colonization and pathogenicity [62,63]. Regulating MAPK activity via protein phosphatases provides critical control during desensitization or adaptation to stimuli. In *S. cerevisiae*, the dual-specificity phosphatase Msg5 dephosphorylates target threonine and tyrosine residues in the MAPKs Mpk1, which regulate cell wall integrity (CWI). *S. cerevisiae* strains lacking the type 2C protein phosphatase Ptc1 and Fusarium oxysporum strains lacking the protein phosphatase Msg5, which target the MAPKK acting upstream of Mpk1, also display increased levels of Mpk1 phosphorylation and sensitivity to cell-wall perturbing compounds [64–66]. In *F. oxysporum*, the msg5 mutant exhibited a 50% reduction in growth compared to the wild type strain. No significant differences were detected in sensitivity to osmotic, oxidative or fungicide stresses [64]. However, low concentrations of hydrogen peroxide promoted the growth of *CfMSG5* deletion mutants, and increased hydrogen peroxide concentrations also significantly reduced the inhibition rate of mutants compared to wild-type and complementation strains. Additionally, an increase in Mpk1 phosphorylation was detected in the mutant; and an increase in Mpk1 phosphorylation was detected in the wild-type following H_2_O_2_ addition (Figure 6h,i). After mutating its activity-related site, the same results were obtained as the *CfMSG5* deletion mutant. It suggested that these results may be related to the dephosphorylation site of the CfMsg5. Taken together, these findings suggest that either the constitutive activation or the disruption of a given MAPK pathway can be detrimental for its biological function, CfMsg5 negatively regulates the MAPK CWI pathway in response to exogenous hydrogen peroxide response by dephosphorylating Mpk1.

This study aimed to unravel the infection mechanism of *Colletotrichum fructicola* on *Camellia oleifera* by identifying key genes associated with leaf lesion size through RNA-seq data. Using Weighted Gene Co-expression Network Analysis (WGCNA), we identified a bZIP transcription factor gene, *CfAP1*, as a hub gene related to leaf lesion size. *CfAP1* contributes to vegetative growth, asexual reproduction, and oxidative stress response in *C. fructicola* and modulates pathogenicity on tea-oil leaves. We discovered that the expression of *CfMSG5*, another key gene, is downregulated in Δ*Cfap1* mutants post H_2_O_2_ treatment. This downregulation, along with CfMsg5’s ring disruption of cell wall integrity (CWI) MAPK pathway inhibition, removal of exogenous reactive oxygen species and ring disruption of unfolded protein response (UPR) pathway inhibition, possibly led to the reduced pathogenicity observed in Δ*Cfmsg5* mutants. In summary, our findings underscore the roles of CfAp1 and CfMsg5 in *C. fructicola* pathogenicity and ROS stress response, shedding light on novel fungal pathogenesis mechanisms and potential therapeutic targets.

## Conclusion

Our study aimed to elucidate the infection mechanism of *Colletotrichum fructicola* on *Camellia oleifera* by identifying key genes associated with leaf lesion size using RNA-seq data from different geographical populations. Utilizing Weighted Gene Co-expression Network Analysis (WGCNA), we identified *CfAP1*, a bZIP transcription factor gene, as a hub gene significantly correlated with leaf lesion size. Functional characterization of *CfAP1* revealed its crucial roles in vegetative growth, asexual reproduction, and oxidative stress response, as well as its significant impact on pathogenicity. The deletion of *CfAP1* (Δ*Cfap1* mutant) led to reduced growth, diminished spore production, lower tolerance to oxidative stress, and impaired pathogenicity, highlighting its role in managing oxidative stress during infection. Additionally, we identified CfMsg5, another key gene whose expression is down-regulated in Δ*Cfap1* mutants under H_2_O_2_ treatment. CfMsg5 is involved in ROS clearance, regulation of the Cell Wall Integrity (CWI) MAPK pathway, and unfolded protein response (UPR), contributing to the pathogenicity of *C. fructicola*. Mutants lacking CfMsg5 exhibited reduced pathogenicity, impaired ROS scavenging, and altered responses to various stresses, suggesting that CfMsg5 negatively regulates the CWI MAPK pathway by dephosphorylating Mpk1. Our findings underscore the essential roles of CfAp1 and CfMsg5 in the pathogenicity and stress response of *C. fructicola*, providing new insights into fungal pathogenesis mechanisms and potential therapeutic targets.

## Materials and methods

### Test strain and growth conditions

The wildtype (WT) strain CFLH16, responsible for anthracnose in tea-oil trees, was isolated from a tea-oil tree field and identified in the China Ministry of Education key laboratory for non-wood forest cultivation and conservation. All strains were cultivated at 28°C in CM. To observe the vegetative growth, CFLH16, mutants and complementation strains were cultivated at 28°C on complete medium (CM) and minimal medium (MM), and diameters of fungal colonies were measured after 3 to 4 days as indicated. To observe the vegetative growth under stress condition, 0.7 M NaCl, 2.5 mm H_2_O_2,_ 5 mm H_2_O_2_, 10 mm H_2_O_2_, 0.5 μg/ml TUNI or 5 mm DTT was mixed in solid CM, and diameters of fungal colonies were measured after 2–3 days as indicated. The study aimed to determine the role of CfAp1 and CfMsg5 proteins in the development and pathogenicity of *C. fructicola* CFLH16.

### Construction of gene co-expression networks (WGCNA)

WGCNA (R package) [22,67] was used to conduct co-expression networks and module detection with powerEstimate 7, maxBlockSize 25,000, minModuleSize 30 and mergeCutHeight 0.25. The module phenotype correlated heatmap was recreated using the local R heatmap package “ggplot2.” Network analysis and visualization was processed with BioSciTools (https://github.com/BioSciTools/BioSciTools.github.io). The function “Network Plot” was processed with the default settings using hub genes in the module “turquoise,” and the GS1 and datKME values of hub genes were greater than 0.94.

### Sequence analysis of C. fructicola Ap1 and Msg5

The Ap1 protein (NP.011987.1) of S. cerevisiae was used as a query to search the *C. fructicola* genome database by BLASTP. The homologous proteins of Ap1 and Msg5 in other species were acquired from the NCBI database. The relationships among Ap1 or Msg5 proteins from different species were constructed by MEGA 7.0 programs using a neighbour-joining method with 1000 bootstrap replicates.

### Deletion and complementation of C. fructicola CfAP1 and CfMSG5

Deletion and complementation of *CfAP1 and CfMSG5* genes were performed using methods referred to Gao et al. [68], and all primers used in this work were listed in Table S1. Using CFLH16 as a template, the upper (approximately one thousand base pairs upstream of the promoter sequence) and lower arms (approximately one thousand base pairs downstream of the terminator sequence) were amplified by corresponding primers 1F/2 R and 3F/4 R, the hygromycin phosphotransferase gene (HPH) fragment (about 1.4 kb) was amplified using Hyg-F/Hyg-R primers. The upper arm, HPH and lower arm were fused by overlap PCR. And then the gene deletion fragment was obtained. The recombinant deletion fragment was transformed into the protoplasts of the wild-type strain of *C. fructicola* by polyethylene glycol (PEG) method. Screening antibiotics for use with hygromycin. Then, the mutant was obtained through 5F/H855R and 7F/8 R primer validation (Figure S2). Primer 5F is located approximately 1500 base pairs upstream of the gene promoter; 7F/8 R constitute a gene-specific primer pair; H855R is a primer located in the HPH.

Using CFLH16 as a template, the promoter and terminator regions of *CfAP1* and *CfMSG5* were amplified by corresponding primers 9F/10 R. These fragments were ligated into pYF11 to obtain plasmid pYF11- *CfMSG5*. Transfer plasmids into mutants by polyethylene glycol (PEG) method to obtain transformants. Screening reagents using bleomycin. Then, the complementation strain was obtained through GFP fluorescence.

### Subcellular localization experiments

The hyphae GFP or RFP fused proteins were observed under fluorescence microscopy (ZEISS, Axio Observer. A1).

CfAp1: H1-RFP were introduced to mark the nuclei. For generating H1-RFP, the ~1.5 kb native promoter and the full-length of H1 was inserted into a pHZ126 vector [13]. Transfer plasmids (pHZ126-H1 and pYF11-*AP1*) into mutants by polyethylene glycol (PEG) method to CFLH16.

CfMsg5: Hyphae of ∆*Cfmsg5*/*CfMSG5* strain were incubated on liquid medium for 24 hours. 7-Amino-4-chloromethylcoumarin (CMAC) staining of vacuole was performed at the 37°C for 30 min.

### Site-directed mutagenesis

The most striking similarity occurs in the region of a conserved cysteine, the so-called HC motif, changing codon Cys319 to Ala319 inactivated the enzyme in vitro [31]. Through sequence analysis, a corresponding amino acid at position 461 was found on the homologous gene *CfMSG5* in *C. fructicola* (Figure 4b). A mutation of *CfMSG5* (∆*Cfmsg5*^Ala461^) containing alanine instead of cysteine at a position 461 was prepared as follows. The first PCR was performed with MSG5-9F prime as the 5’primer and the 3’ primer MSG5^Ala416^-R. The second PCR was performed with the 5” primer MSG5^Ala416^-F and MSG5-10 R primer as the 3” primer. Both PCRs used CFLH16 as the template. These two digested fragments were ligated into pYF11 to obtain pYF11-*CfMSG5*^Ala461^. Transfer plasmids into mutants to obtain point mutation mutants transformants. Screening reagents using bleomycin. Then, the mutant was obtained through GFP fluorescence screening and MSG5-5F/H855R and MSG5-7F/MSG5-8 R primer validation (Figure S2).*Asexual* Conidia, Appressorium Formation and collapse rate Assays

The mycelia of CFLH16, mutants, and complementation strains were cultured in liquid shaking CM for two days medium at 28°C and 180 rpm, and then the conidiation was quantitatively determined and spared for subsequent experiments. Appressorium formation and collapse rate assays were conducted by incubating the spore suspension of CFLH16, the mutant, and complementation strains on the centre of hydrophobic slides at 28°C for 24 h, and then statistically analysing the rates of appressorium formation. The appressorium was soaked in 20 μl glycerol(2 mol/L), and the collapse rate was calculated after 10 minutes.

### Pathogenicity assays, DAB staining and H_2_O_2_ content determination

Pathogenicity assays were performed by inoculating mycelial blocks of the mutant and complementation strains (Φ = 6 mm) onto the abaxial edge of unwounded or wounded tea-oil tree leaves and incubating them in darkness at 28°C with 100% humidity for 2–4 days, followed by measuring the sizes of the lesions.

DAB staining method referenced Yu et al. [69] and used plant hydrogen peroxide staining solution (DAB) (Servicebio, G1022). Adjust the conidia concentration to 10^6^ conidia/ml, spray onto fresh tea-oil tree leaves using a watering can, and spray with sterile water as a blank control. Incubating them in darkness at 28°C with 100% humidity for 1–4 days. Specifically, the leaves were immersed in DAB operating fluid (1 mg/mL DAB in phosphate buffer). Samples were vacuum-infiltrated for 1 h and then incubated for 5 h at 28°C , with gentle shaking at 70 rpm. Subsequently, samples were 95% ethanol and incubated at 40°C until complete bleach. The lesion is cut for observation and pictures were taken under a light microscope. The H_2_O_2_ content in the leaf was detected by visible spectrophotometry, and the hydrogen peroxide (H_2_O_2_) content detection kit (Beijing Solarbio Science & Technology Co., Ltd, BC3590) was used.

### Rna-seq analysis

Wildtype and the Δ*Cfap1* mutant strains were cultured in 100 ml PDA medium at 28°C for 1 day, and a final concentration of 2.5 mm of H_2_O_2_ was added at 28°C for another day and sent to the Major company for RNA sequencing analysis. The raw RNA sequencing data was obtained from NCBI Sequencing Read Archive (SRA, https://www.ncbi.nlm.nih.gov/sra) through the SRA toolkits “prefetch” (version 2.8.0), Project “PRJNA644240” was downloaded for WGCNA analysis [24]. Raw data (raw reads) in fastq format were first qualified with FastQC program (https://www.bioinformatics.babraham.ac.uk/projects/fastqc/) for Q20, Q30, GC-content and sequence duplication level; the data was then processed in Hisat2 version 2.2.1 [70] for read alignment to the *C. fructicola* CFLH16. The reads were subjected to fragments per kilobase of transcript per million fragments mapped (FPKM) conversion to obtain the expression value of genes and transcripts. In-house R scripts were used to analyse gene expression and generate heatmaps. Heatmap was generated using ggplot2, reshape2, gplots, and dplyr packages in R version 4.1.2. All of the raw data from the RNA-seq are available from the SRA database with accession numbers SRR26935071, SRR26935070, and SRR26935069 for WT 1 to 3 and SRR26935068, SRR26935067, and SRR26935066 for ∆*Cfap1* 1 to 3.

### Quantitative real‑time PCR

Initially, 200 μl of spore suspension (5 × 10^4^ conidia/ml) was spread onto cellophane membrane-covered CM plates and incubated for two days. Aerial mycelia were harvested, and total RNA was isolated using the Trizol reagent as per the manufacturer’s guidelines (TaKaRa, Japan). The RNA was then reverse transcribed into cDNA with the PrimeScript™ RT reagent kit with gDNA Eraser (TaKaRa, Japan). Gene expression analysis was carried out using quantitative Real-Time PCR (qPCR) on a Mastercycler Real-Time PCR Detection System (Eppendorf, Germany). This was performed using the SYBR Premix Ex Taq (Tli RNaseH Plus) kit (TaKaRa, Japan) according to the manufacturer’s instructions. The average threshold cycle (CT) was normalized to the housekeeping genes, 40S and α-ACTIN, for each strain, with relative transcript abundance calculated as 2^-∆CT, where ∆CT = CT_gene – (CT_40S + CT_Actin)/2. Fold changes in gene expression between two strains were determined by calculating ∆∆CT values as ∆∆CT = ∆CT_strain_1 – ∆CT_strain_2, and converted into fold changes using the formula 2^-∆∆CT, as detailed by Livak and Schmittgen [71]. The primers used in this study for PCR are listed in Table S1.

### Analysis of MAPK phosphorylation via western blot

To examine the impact of Msg5 on MAPK phosphorylation, mycelia from CFLH16, mutant, and complementation strains were cultured in a liquid shaking CM medium at 28°C and 180 rpm for a duration of 24 hours. Mycelium samples were collected before (time 0) and 30 minutes post the addition of 5 mm H_2_O_2_. Total protein was extracted using RIPA lysis buffer and further treated with a phosphatase inhibitor cocktail (100×), protease inhibitor cocktail (100×, EDTA-free), and Phenyl methane sulphonyl fluoride (PMSF), subsequently placed on ice for 30 minutes with stirring at 10-minute intervals. The Western blot procedure was carried out as detailed by Nordzieke et al. [72]. Briefly, protein samples were run on 7.5% SDS-polyacrylamide gels (Omni-Easy™One-Step PAGE Gel Fast Preparation Kit, PG211) and transferred onto a nitrocellulose membrane using the wet transfer method. Phosphorylation of Mpk1 and Fmk1 MAPKs was detected with the Phospho-ERK1/2 rabbit mAb antibody ((Thr202/Tyr204)/(Thr185/Tyr187); Zen bio; cat#R24245) [64]. Beta tubulin (6D8) mouse mAb antibody (Zen bio; cat#250007) served as a loading control. The hybridized bands were visualized using the ECL hypersensitive chemiluminescence solution (Zen bio, cat#17045) in a Tanon automatic chemiluminescence imager. Densitometric quantification of western blot signals was carried out using the ImageJ software, as described [72]. The ratio of phospho-MAPK to Beta tubulin signal was determined and normalized to the wildtype level under non-inducing conditions (time 0) for each individual blot. The experiment was repeated thrice. Statistical analyses were conducted using the t-test for unequal variances, also known as Welch’s test.

## Supplementary Material

Supplemental Material

Caption_240673762.docx

## Data Availability

The data supporting the findings of this study are available on ScienceDB https://www.scidb.cn/en/s/JNRBf2.

## References

[1] Zhao J, Lu Z, Wang L, et al. Plant responses to heat stress: physiology, transcription, noncoding RNAs, and epigenetics. Int J Mol Sci. 2020;22(1):117. doi: 10.3390/ijms22010117 33374376 PMC7795586

[2] Lawas LM, Zuther E, Jagadish S, et al. Molecular mechanisms of combined heat and drought stress resilience in cereals. Curr Opin Plant Biol. 2018;45:212–20. doi: 10.1016/j.pbi.2018.04.002 29673612

[3] Nejat N, Mantri N. Plant immune system: crosstalk between responses to biotic and abiotic stresses the missing link in understanding plant defence. Curr Issues Mol Biol. 2017;23:1–16. doi: 10.21775/cimb.023.001 28154243

[4] Fisher MC, Henk DA, Briggs CJ, et al. Emerging fungal threats to animal, plant and ecosystem health. Nature. 2012;484(7393):186–194. doi: 10.1038/nature10947 22498624 PMC3821985

[5] Chen JM, Yang X, Huang X, et al. Leaf transcriptome analysis of a subtropical evergreen broadleaf plant, wild oil-tea camellia (*Camellia oleifera*), revealing candidate genes for cold acclimation. BMC Genomics. 2017;18(1):211. doi: 10.1186/s12864-017-3570-4 28241790 PMC5329932

[6] Peng S, Lu J, Zhang Z, et al. Global transcriptome and correlation analysis reveal cultivar-specific molecular signatures associated with fruit development and fatty acid determination in *Camellia oleifera* Abel. Int J Genomics. 2020;2020:1–16. doi: 10.1155/2020/6162802 PMC7481963 32953873

[7] He Z, Liu C, Zhang Z, et al. Integration of mRNA and miRNA analysis reveals the differentially regulatory network in two different *Camellia oleifera* cultivars under drought stress. Front Plant Sci. 2022;13:1001357. doi: 10.3389/fpls.2022.1001357 36247533 PMC9562160

[8] Jin AX, Zhou GY, Li H. Progress, problem and prospect of oil *camelliae* anthracnose (*Colletotrichum gloeosporioides*) research. For Pest Dis. 2009;28:27–31.

[9] Wang X, Zhao C, Müller C, et al. Emergent constraint on crop yield response to warmer temperature from field experiments. Nat Sustainability. 2020;3(11):908–916. doi: 10.1038/s41893-020-0569-7

[10] Huang L, Li QC, Zhang Y, et al. *Colletotrichum gloeosporioides* sensu stricto is a pathogen of leaf anthracnose on evergreen spindle tree (*Euonymus japonicus*). Plant Dis. 2016;100(12):2531–2541. doi: 10.1094/PDIS-07-15-0740-RE 30688606

[11] Li H, Zhou GY, Liu JA, et al. Population genetic analyses of the fungal pathogen *Colletotrichum fructicola* on tea-oil trees in China Papa, R. (Ed.). PLoS One. 2016;11(6):e0156841. doi: 10.1371/journal.pone.0156841 27299731 PMC4907445

[12] Yao Q, Guo Y, Wei F, et al. A bZIP-type transcription factor CfHac1 is involved in regulating development and pathogenesis in *Colletotrichum fructicola*. Mycosystema. 2019;38:1643–1652. doi: 10.13346/j.mycosystema.190228

[13] Zhang SP, Guo Y, Li SZ, et al. Histone acetyltransferase CfGcn5-mediated autophagy governs the pathogenicity of *Colletotrichum fructicola*. MBio. 2022;13(5):e01956–22. doi: 10.1128/mbio.01956-22 35975920 PMC9600425

[14] Zhang SP, Guo Y, Li SP, et al. Functional analysis of CfSnf1 in the development and pathogenicity of anthracnose fungus *Colletotrichum fructicola* on tea-oil tree. BMC Genet. 2019;20(1):94. doi: 10.1186/s12863-019-0796-y 31805867 PMC6896739

[15] Camejo D, Guzmán-Cedeño Á, Moreno A. Reactive oxygen species, essential molecules, during plant–pathogen interactions. Plant Physiol Biochem. 2016;103:10–23. doi: 10.1016/j.plaphy.2016.02.035 26950921

[16] Segal LM, Wilson RA. Reactive oxygen species metabolism and plant-fungal interactions. Fungal Genet Biol. 2018;110:1–9. doi: 10.1016/j.fgb.2017.12.003 29225185

[17] Boyd LA, Ridout C, O’Sullivan DM, et al. Plant–pathogen interactions: disease resistance in modern agriculture. Trends Genet. 2013;29(4):233–240. doi: 10.1016/j.tig.2012.10.011 23153595

[18] Miya A, Albert P, Shinya T, et al. CERK1, a LysM receptor kinase, is essential for chitin elicitor signaling in *Arabidopsis*. Proc Natl Acad Sci USA. 2007;104(49):19613–19618. doi: 10.1073/pnas.0705147104 18042724 PMC2148337

[19] Wang T, Gasciolli V, Gaston M, et al. LysM receptor-like kinases involved in immunity perceive lipo-chitooligosaccharides in mycotrophic plants. Plant Physiol. 2023;192(2):1435–1448. doi: 10.1093/plphys/kiad059 36722175 PMC10231384

[20] Tang Z, Cai S, Liu Y, et al. A lipopolysaccharide O-antigen synthesis gene in *Mesorhizobium huakuii* plays differentiated roles in root nodule symbiotic compatibility with *Astragalus sinicus*. Mol Plant-Microbe Interact. 2023;36(10):623–635. doi: 10.1094/MPMI-05-23-0066-R 37366577

[21] Roudaire T, Marzari T, Landry D, et al. The grapevine LysM receptor-like kinase VvLYK5-1 recognizes chitin oligomers through its association with VvLYK1-1. Front Plant Sci. 2023;14:14. doi: 10.3389/fpls.2023.1130782 PMC9932513 36818830

[22] Langfelder P, Horvath S. WGCNA: an R package for weighted correlation network analysis. BMC Bioinformatics. 2008;9(1):559. doi: 10.1186/1471-2105-9-559 19114008 PMC2631488

[23] Kumar K, Kumar M, Kim SR, et al. Insights into genomics of salt stress response in rice. Rice. 2019;12(1):1–15. doi: 10.1186/1939-8433-6-27 24280112 PMC4883734

[24] Tan S, Chen Y, Zhou G, et al. Transcriptome analysis of *Colletotrichum fructicola* infecting *Camellia oleifera* indicates that two distinct geographical fungi groups have different destructive proliferation capacities related to purine metabolism. Plants. 2021;10(12):2672. doi: 10.3390/plants10122672 34961144 PMC8708221

[25] Guo M, Chen Y, Du Y, et al. The bZIP transcription factor MoAP1 mediates the oxidative stress response and is critical for pathogenicity of the rice blast fungus *Magnaporthe oryzae*. PloS Pathog. 2011;7(2):e1001302. doi: 10.1371/journal.ppat.1001302 21383978 PMC3044703

[26] Nguyen AN, Lee A, Place W, et al. Multistep phosphorelay proteins transmit oxidative stress signals to the fission yeast stress-activated protein kinase. Mol Biol Cell. 2000;11(4):1169–1181. doi: 10.1091/mbc.11.4.1169 10749922 PMC14839

[27] Quinn J, Findlay VJ, Dawson K, et al. Distinct regulatory proteins control the graded transcriptional response to increasing H_2_O_2_ levels in fission yeast *Schizosaccharomyces pombe*. Mol Biol Cell. 2002;13(3):805–816. doi: 10.1091/mbc.01-06-0288 11907263 PMC99600

[28] Chi MH, Park SY, Kim S, et al. A novel pathogenicity gene is required in the rice blast fungus to suppress the basal defenses of the Host Howlett B.J. (Ed.). PLOS Pathog. 2009;5(4):e1000401. doi: 10.1371/journal.ppat.1000401 19390617 PMC2668191

[29] Lin CH, Yang SL, Chung KR. The YAP1 homolog–mediated oxidative stress tolerance is crucial for pathogenicity of the necrotrophic fungus *Alternaria alternata* in citrus. Mol Plant-Microbe Interact. 2009;22(8):942–952. doi: 10.1094/MPMI-22-8-0942 19589070

[30] Molina L, Kahmann R. An ustilago maydis gene involved in H_2_O_2_ detoxification is required for virulence. Plant Cell. 2007;19(7):2293–2309. doi: 10.1105/tpc.107.052332 17616735 PMC1955693

[31] Doi K, Gartner A, Ammerer G, et al. MSG5, a novel protein phosphatase promotes adaptation to pheromone response in *S. cerevisiae*. Embo J. 1994;13(1):61–70. doi: 10.1002/j.1460-2075.1994.tb06235.x 8306972 PMC394779

[32] Fisher EH, Charbonneau H, Tonks NK. Protein tyrosine phosphatases: a diverse family of intracellular and transmembrane enzymes. Science. 1991;253(5018):401–406. doi: 10.1126/science.1650499 1650499

[33] Streuli M, Krueger NX, Thai T, et al. Distinct functional roles of the two intracellular phosphatase like domains of the receptor-linked protein tyrosine phosphatases LCA and LAR. Embo J. 1990;9(8):2399–2407. doi: 10.1002/j.1460-2075.1990.tb07415.x 1695146 PMC552264

[34] Guan KL, Broyles SS, Dixon JE. A Tyr/Ser protein phosphatase encoded by vaccinia virus. Nature. 1991;350(6316):359–362. doi: 10.1038/350359a0 1848923

[35] Dodds PN, Rafiqi M, Gan PHP, et al. Effectors of biotrophic fungi and oomycetes: pathogenicity factors and triggers of host resistance. New Phytol. 2009;183(4):993–1000. doi: 10.1111/j.1469-8137.2009.02922.x 19558422

[36] Hogenhout SA, Van der Hoorn RAL, Terauchi R, et al. Emerging concepts in effector biology of plant-associated organisms. Mol Plant-Microbe Interact. 2009;22(2):115–122. doi: 10.1094/MPMI-22-2-0115 19132864

[37] Kuhn K, Ralp P. Introduction to a *virtual special issue* on phytopathogen effectorproteins. New Phytol. 2014;202(3):727–730. doi: 10.1111/nph.12804 24716512

[38] Apel K, Hirt H. REACTIVE OXYGEN SPECIES: metabolism, oxidative stress, and signal transduction. Annu Rev Plant Biol. 2004;55(1):373–399. doi: 10.1146/annurev.arplant.55.031903.141701 15377225

[39] Hammond-Kosack KE, Jones JD. Resistance gene-dependent plant defense responses. Plant Cell. 1996;8(10):1773–1791. doi: 10.1105/tpc.8.10.1773 8914325 PMC161314

[40] Tenhake R, Levine A, Brisson LF, et al. Function of the oxidative burst in hypersensitive disease resistance. Proc Natl Acad Sci USA. 1995; 92(10): 4158–4163. doi: 10.1073/pnas.92.10.4158 11607542 PMC41903

[41] Torres MA, Jones JDG, Dangl JL. Pathogen-induced, NADPH oxidase–derived reactive oxygen intermediates suppress spread of cell death in Arabidopsis thaliana. Nat Genet. 2005;37(10):1130–1134. doi: 10.1038/ng1639 16170317

[42] Kang J, Brajanovski N, Chan KT, et al. Ribosomal proteins and human diseases: molecular mechanisms and targeted therapy. Signal Transduct Target Ther. 2021;6(1):323. doi: 10.1038/s41392-021-00728-8 34462428 PMC8405630

[43] Hong SY, Roze LV, Linz JE. Oxidative stress-related transcription factors in the regulation of secondary metabolism. Toxins (Basel). 2013;5(4):683–702. doi: 10.3390/toxins5040683 23598564 PMC3705287

[44] Reverberi M, Zjalic S, Ricelli A, et al. Modulation of antioxidant defense in *Aspergillus parasiticus* is involved in aflatoxin biosynthesis: a role for the ApyapA gene. Eukaryot Cell. 2008;7(6):988–1000. doi: 10.1128/EC.00228-07 18441122 PMC2446656

[45] Toone WM, Jones N. Stress-activated signalling pathways in yeast. Genes Cell. 1998;3(8):485–498. doi: 10.1046/j.1365-2443.1998.00211.x 9797451

[46] Wu AL, Moye-Rowley WS. GSH1, which encodes gamma-glutamylcysteine synthetase, is a target gene for yAP-1 transcriptional regulation. Mol Cell Biol. 1994;14(9):5832–5839. doi: 10.1128/MCB.14.9.5832 7915005 PMC359109

[47] Zhan XL, Deschenes RJ, Guan KL. Differential regulation of FUS3 MAP kinase by tyrosine-specific phosphatases *PTP2/PTP3* and dual-specificity phosphatase *MSG5* in *Saccharomyces cerevisiae*. Genes & Devel. 1997;11(13):1690–1702. doi: 10.1101/gad.11.13.1690 9224718

[48] Andersson J, Simpson DM, Qi M, et al. Differential input by Ste5 scaffold and Msg5 phosphatase route a MAPK cascade to multiple outcomes. Embo J. 2004;23(13):2564–2576. doi: 10.1038/sj.emboj.7600250 15192700 PMC449765

[49] Flández M, Cosano IC, Nombela C, et al. Reciprocal regulation between Slt2 MAPK and isoforms of Msg5 dual-specificity protein phosphatase modulates the yeast cell integrity pathway. J Biol Chem. 2004;279(12):11027–11034. doi: 10.1074/jbc.M306412200 14703512

[50] Martín H, Rodríguez-Pachón JM, Ruiz C, et al. Regulatory mechanisms for modulation of signaling through the cell integrity Slt2-mediated pathway in *Saccharomyces cerevisiae*. J Biol Chem. 2000;275(2):1511–1519. doi: 10.1074/jbc.275.2.1511 10625705

[51] Wurgler-Murphy SM, Maeda T, Witten EA, et al. Regulation of the *Saccharomyces cerevisiae* HOG1 mitogen-activated protein kinase by the PTP2 and PTP3 protein tyrosine phosphatases. Mol Cell Biol. 1997;17(3):1289–1297. doi: 10.1128/MCB.17.3.1289 9032256 PMC231854

[52] Yi M, Chi MH, Khang CH, et al. The ER chaperone LHS1 is involved in asexual development and rice infection by the blast fungus *Magnaporthe oryzae*. Plant Cell. 2009;21(2):681–695. doi: 10.1105/tpc.107.055988 19252083 PMC2660637

[53] Roth G, Vanz AL, Lünsdorf H, et al. Fate of the UPR marker protein Kar2/Bip and autophagic processes in fed-batch cultures of secretory insulin precursor producing pichia pastoris. Microb Cell Fact. 2018;17(1):123. doi: 10.1186/s12934-018-0970-3 30092809 PMC6083527

[54] Tang W, Ru Y, Hong L, et al. System-wide characterization of bZIP transcription factor proteins involved in infection-related morphogenesis of *Magnaporthe oryzae*. Environ Microbiol. 2015;17(4):1377–1396. doi: 10.1111/1462-2920.12618 25186614 PMC4362868

[55] Nakatsukasa K, Kamura T, Brodsky JL. Recent technical developments in the study of er-associated degradation. Curr Opin Cell Biol. 2014;29:82–91. doi: 10.1016/j.ceb.2014.04.008 24867671 PMC4130770

[56] Santamaría PG, Mazón MJ, Eraso P, et al. UPR: an upstream signal to EMT induction in cancer. J Clin Med. 2019;8(5):624. doi: 10.3390/jcm8050624 31071975 PMC6572589

[57] Beaupere C, Labunskyy VM. (Un)folding mechanisms of adaptation to ER stress: lessons from aneuploidy. Curr Genet. 2019;65(2):467–471. doi: 10.1007/s00294-018-0914-9 30511161 PMC6421085

[58] Freedman RB, Hirst TR, Tuite MF. Protein disulphide isomerase: building bridges in protein folding. Trends Biochem Sci. 1994;19(8):331–336. doi: 10.1016/0968-0004(94)90072-8 7940678

[59] Frand AR, Kaiser CA. Ero1p oxidizes protein disulfide isomerase in a pathway for disulfide bond formation in the endoplasmic reticulum. Mol Cell. 1999;4(4):469–477. doi: 10.1016/S1097-2765(00)80198-7 10549279

[60] Krysan DJ. The cell wall and endoplasmic reticulum stress responses are coordinately regulated in *Saccharomyces cerevisiae*. Communicative & Intgr Biol. 2009;2(3):233–235. doi: 10.4161/cib.2.3.8097 PMC2717530 19641740

[61] Turrà D, Segorbe D, Di Pietro A. Protein kinases in plant-pathogenic fungi: conserved regulators of infection. Annu Rev Phytopathol. 2014;52(1):267–288. doi: 10.1146/annurev-phyto-102313-050143 25090477

[62] Segorbe D, Di Pietro A, Pérez-Nadales E, et al. Three *Fusarium oxysporum* mitogen-activated protein kinases (MAPKs) have distinct and complementary roles in stress adaptation and cross-kingdom pathogenicity. Mol Plant Pathol. 2017;18(7):912–924. doi: 10.1111/mpp.12446 27301316 PMC6638227

[63] Turrà D, El Ghalid M, Rossi F, et al. Fungal pathogen uses sex pheromone receptor for chemotropic sensing of host plant signals. Nature. 2015;527(7579):521–524. doi: 10.1038/nature15516 26503056

[64] Fernandes TR, Sánchez Salvador E, Tapia ÁG, et al. Dual-specificity protein phosphatase Msg5 controls cell wall integrity and virulence in *Fusarium oxysporum*. Fungal Genet Biol. 2021;146:103486. doi: 10.1016/j.fgb.2020.103486 33232812

[65] Sharmin D, Sasano Y, Sugiyama M, et al. Effects of deletion of different PP2C protein phosphatase genes on stress responses in *Saccharomyces cerevisiae*. Yeast. 2014;31(10):393–409. doi: 10.1002/yea.3032 25088474

[66] Tatjer L, Sacristán-Reviriego A, Casado C, et al. Wide-ranging effects of the yeast Ptc1 protein phosphatase acting through the MAPK kinase Mkk1. Genetics. 2016;202(1):141–156. doi: 10.1534/genetics.115.183202 26546002 PMC4701081

[67] Zhang B, Horvath S. A general framework for weighted gene co-expression network analysis. Stat Appl Genet Mol Biol. 2005;4(1). doi: 10.2202/1544-6115.1128 16646834

[68] Gao Y, Zhang S, Li H. H3K4 methyltransferase CfSet1 is required for development and pathogenesis in *Colletotrichum fructicola*. J Fungi. 2022;8(4):363. doi: 10.3390/jof8040363 PMC9025643 35448594

[69] Yu X, Xie Y, Luo D, et al. A phospho-switch constrains BTL2-mediated phytocytokine signaling in plant immunity. Cell. 2023;186(11):2329–2344.e20. doi: 10.1016/j.cell.2023.04.027 37192618 PMC10281528

[70] Kim D, Langmead B, Salzberg SL. HISAT: a fast spliced aligner with low memory requirements. Nat Methods. 2015;12(4):357–360. doi: 10.1038/nmeth.3317 25751142 PMC4655817

[71] Livak KJ, Schmittgen TD. Analysis of relative gene expression data using real-time quantitative PCR and the 2−ΔΔCT method. Methods. 2001;25(4):402–408. doi: 10.1006/meth.2001.1262 11846609

[72] Nordzieke DE, Fernandes TR, El Ghalid M, et al. NADPH oxidase regulates chemotropic growth of the fungal pathogen *Fusarium oxysporum* towards the host plant. New Phytol. 2019;224(4):1600–1612. doi: 10.1111/nph.16085 31364172

