# Supplementary figures and images for: A Colletotrichum fructicola dual specificity phosphatase CfMsg5 is regulated by the CfAp1 transcription factor during oxidative stress and promotes virulence on Camellia oleifera
Source: Virulence. 2024 Oct 18;15(1):2413851. doi: 10.1080/21505594.2024.2413851 (PMC11492636; doi:10.1080/21505594.2024.2413851)

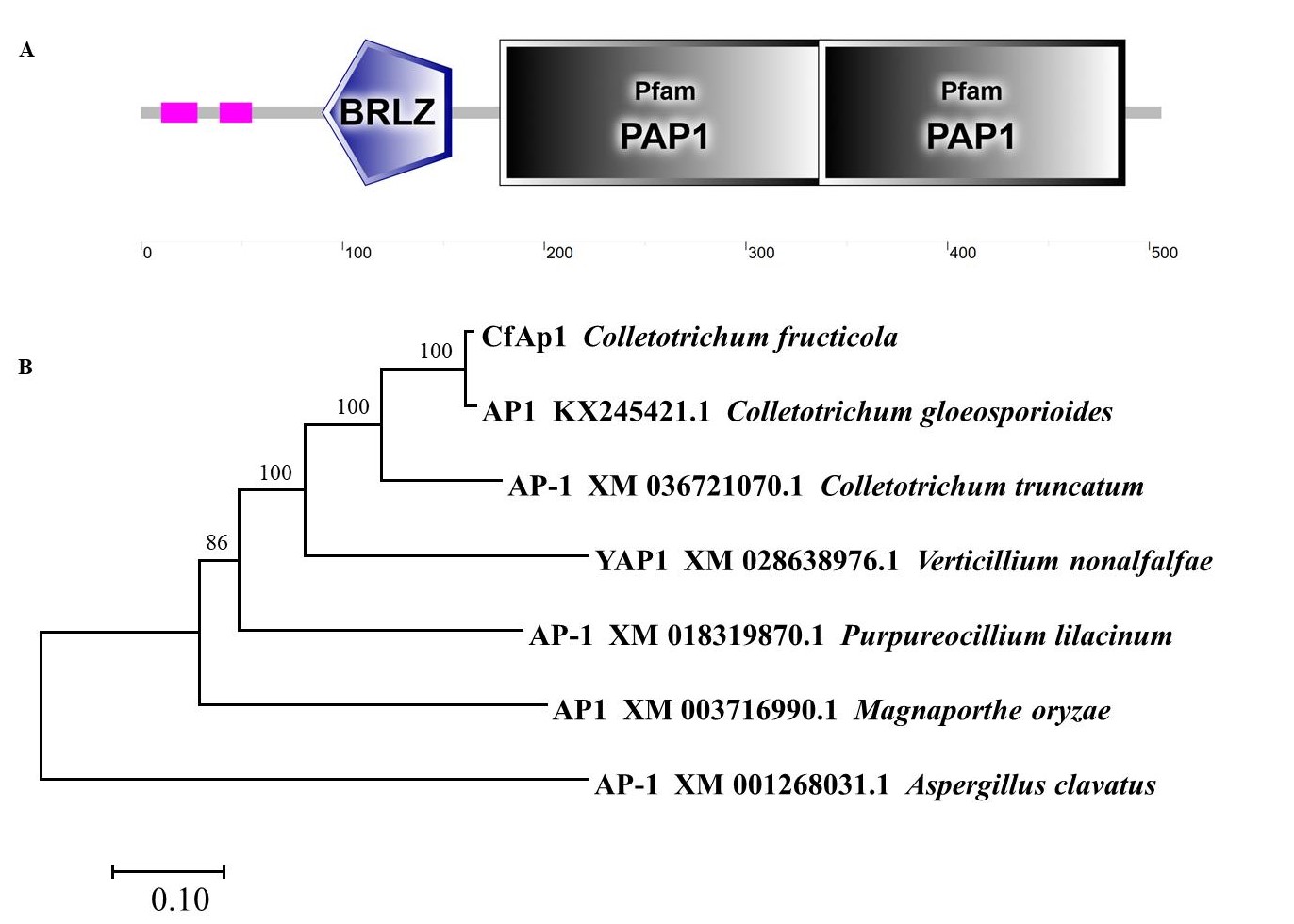

Supplement: Supplemental Material [file KVIR_A_2413851_SM1803.zip › supp fig 1.jpeg]

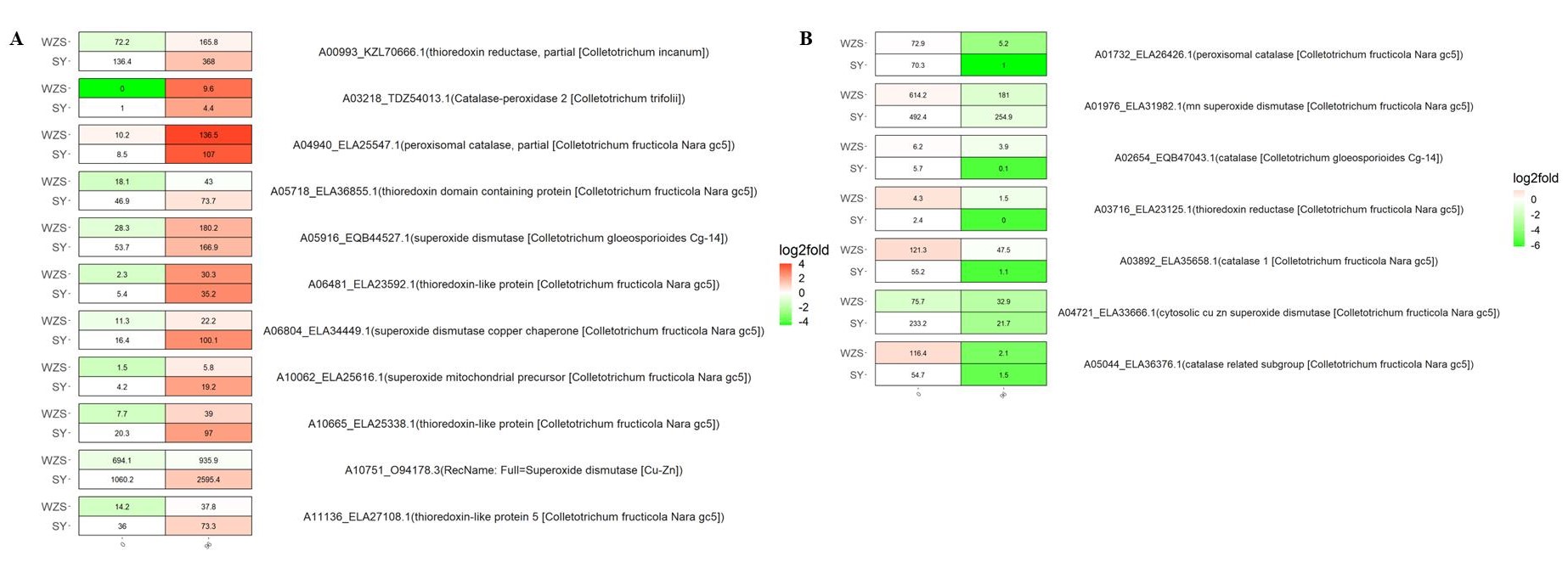

Supplement: Supplemental Material [file KVIR_A_2413851_SM1803.zip › supp fig 2.jpeg]

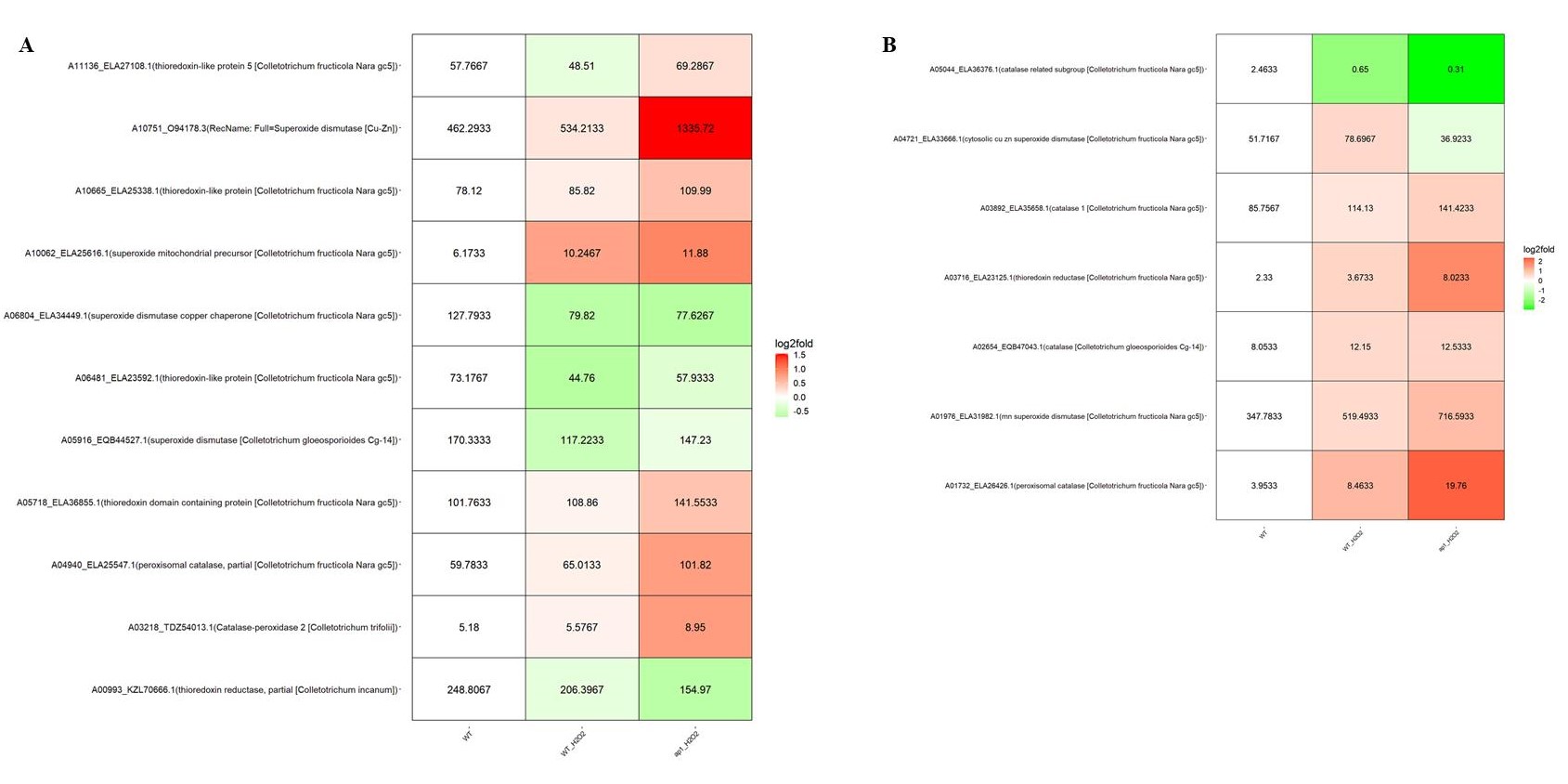

Supplement: Supplemental Material [file KVIR_A_2413851_SM1803.zip › supp fig 3.jpeg]

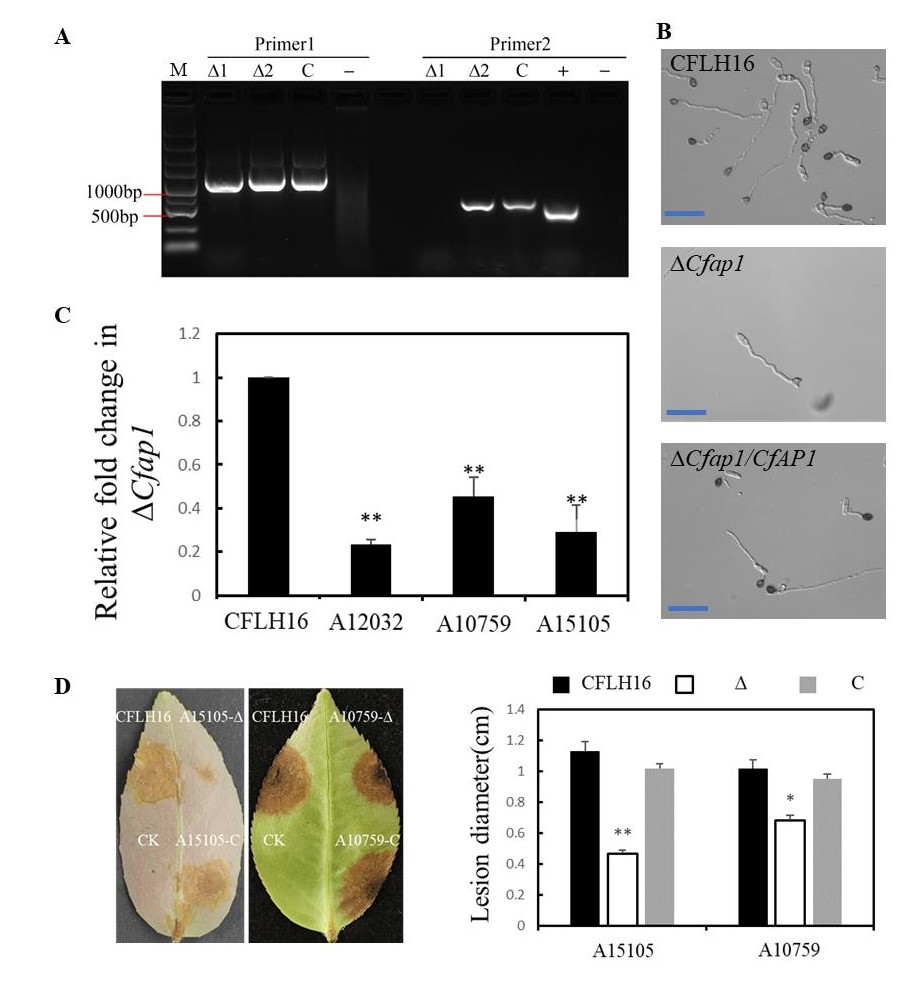

Supplement: Supplemental Material [file KVIR_A_2413851_SM1803.zip › supp fig 4.jpeg]

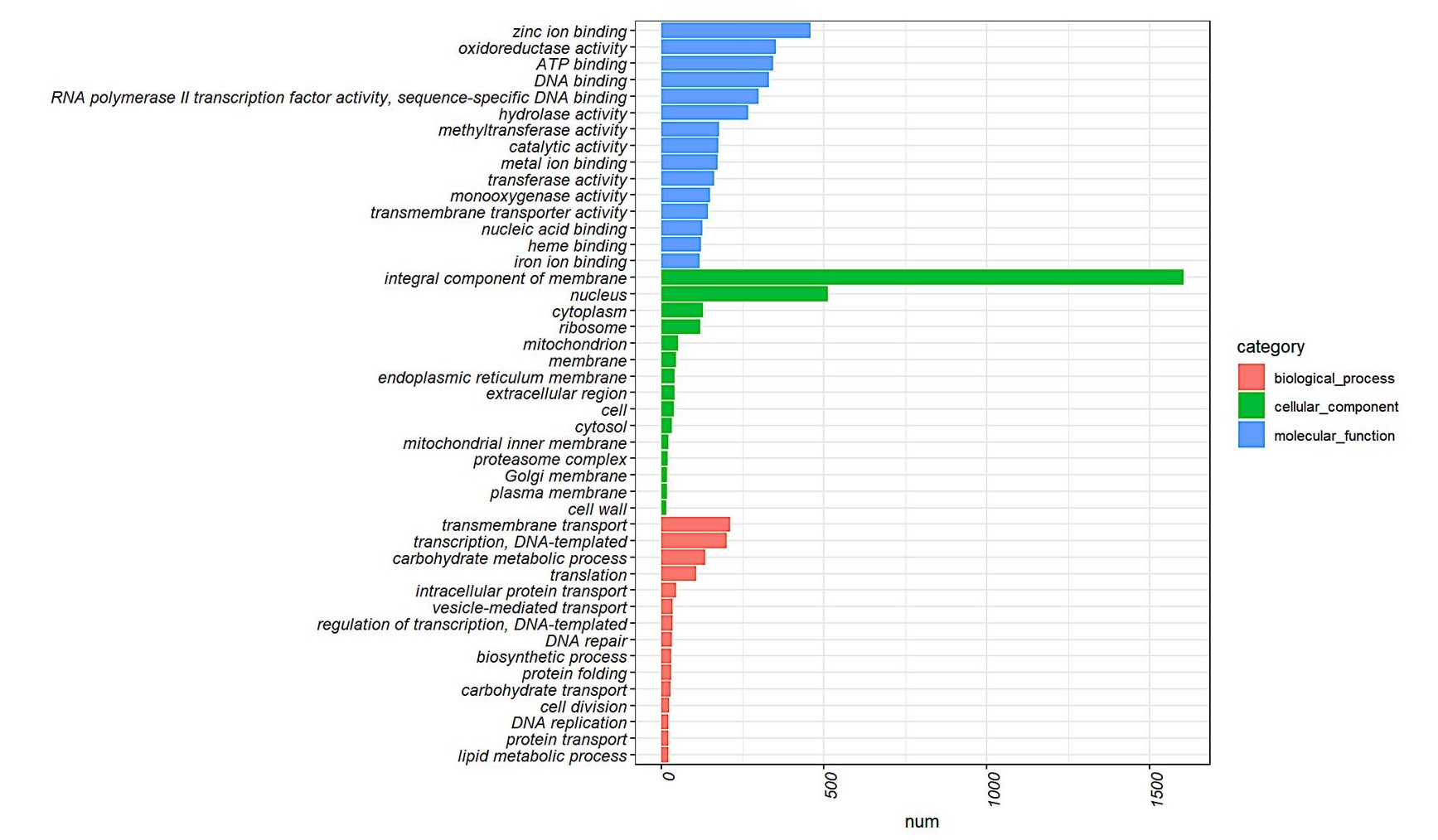

Supplement: Supplemental Material [file KVIR_A_2413851_SM1803.zip › supp fig 5.jpeg]
