# Supplementary material for: A Colletotrichum fructicola dual specificity phosphatase CfMsg5 is regulated by the CfAp1 transcription factor during oxidative stress and promotes virulence on Camellia oleifera
Source: Virulence. 2024 Oct 18;15(1):2413851. doi: 10.1080/21505594.2024.2413851 (PMC11492636; doi:10.1080/21505594.2024.2413851)
Supplement: Caption_240673762.docx [file KVIR_A_2413851_SM6690.docx]

**Figures**

Fig. 1. WGCNA to identify hub genes during infection

A. WGCNA module-phenotypes correlation analysis B. Module membership in turquoise module. C. Networks of hub genes in module turquoise, D. Heatmap of hub genes in modules during infection of *C. fructicola* on oil-tea leaves. The heatmap represents the expression pattern of genes in response to cold stress. Relative expression levels were calculated as a Log2-fold change against CK (see Section Materials and Methods). The red color shows an upregulation of a given gene, and the green indicates a downregulation. Labeled number in each tile is the expression level “FPKM”.

Fig. 2 The loss of *CfAP1* led to the weakening of the pathogenicity of *C. fruitiflora*.

A. The hyphae of the mutant strain were sparse and loose, while those of the wildtype strain were tightly intertwined. B. The growth of the wildtype strain (CFLH16), the Δ*Cfap1* mutant strain, and the Δ*Cfap1*/*CfAP1* complemented strain on CM with 0/5/10 mM H_2_O_2_ at 28℃ in the dark for 2 days. C. Statistical analysis of the difference in colony diameter among the CFLH16, the Δ*Cfap1* mutant strain, and the Δ*Cfap1*/*CfAP1* complemented strain on CM (**: P<0.01). D. Statistical analysis of the growth inhibition rate of the strains under H_2_O_2_ stress (**: P<0.01). E. The lesions caused by the CFLH16, the Δ*Cfap1* mutant strain, and the Δ*Cfap1*/*CfAP1*complemented strain on unwound tea leaves. F. Statistical analysis of the difference in lesion diameter (**: P<0.01). G. Statistical analysis of the conidiation (2 days) of CFLH16, the Δ*Cfap1* mutant strain, and the Δ*Cfap1*/*CfAP1* complemented strain. H. Statistical analysis of the appressorium formation rate (24 h) of the CFLH16, the Δ*Cfap1* mutant strain, and the Δ*Cfap1*/*CfAP1* complemented strain. Statistical analysis of the rate of appressorium formation on hydrophobic slides. I. Statistical analysis of the rate of appressorium formation collapse (**: P<0.01).

Fig. 3. Candidate genes selected from AP1 H_2_O_2_ treatment RNA-seq data.

The heatmap represents the expression pattern of genes in response to cold stress. Relative expression levels were calculated as a Log2-fold change against CK (see Section Materials and Methods). The red color shows an upregulation of a given gene, and the green indicates a downregulation. Labeled number in each tile is the expression level “FPKM”.

Fig. 4 CfMsg5 capital domain and phylogenetic analysis

A. The CfMsg5 capital domain was found using the SMART website (http://smart.embl-heidelberg.de/); DSPc: Dual specificity phosphatase, catalytic domain; the number denotes the amino acid's serial number，pink boxes represent an unknown domain. B. Amino acid sequence alignment of DSPc domain of Colletotrichum fructicola Msg5, Colletotrichum aenigma pmp1, Colletotrichum gloeosporioides Msg5, Colletotrichum spinosum Pmp1, *Verticillium longisporum* Pmp1, *Plectosphaerella plurivora* Pmp1, *Plectosphaerella cucumerina* Msg5 and *Saccharomyces cerevisiae* Msg5. Red background indicates the residues that are completely identical. The conserved amino acid residues are boxed. Dashes are inserted to allow for maximal alignment of the sequences. Asterisks and numbers represent mutation sites. C. The phylogenetic tree was built using the neighbor-joining (NJ) technique and analyzed in Mega7.0 with 1,000 bootstrap repetitions; the number on the branch represents the support determined with 100 bootstrap replicates in the NJ analysis; the scale bar represents the branch length. The number in front of each reference taxon is the GenBank accession number of the orthologous amino acid sequence to CfMsg5.

Fig. 5 The pathogenicity of *C. fructicola* was regulated by CfMsg5.

A. H1 was used as a nucleus marker. The colocalization of CfAp1-GFP and H1-RFP showed that CfAp1 localized to the nucleus in hyphae. Hyphae of ∆*Cfmsg5*/*CfMSG5* strain were incubated on liquid medium for 24 h.7-Amino-4-chloromethylcoumarin (CMAC) staining of vacuole was performed at the 37°C for 30 min. CfMsg5 is distributed evenly throughout the cytoplasm of mature hyphae without localization to the vacuole membrane. Bar = 10 um.

B. The lesions caused by the wildtype strain (CFLH16), the Δ*Cfmsg5* mutant strain, and the complemented strain on wounded tea-oil leaves. C. The appressorium of the wildtype strain (CFLH16), the Δ*Cfmsg5* mutant strain, and the ∆*Cfmsg5*/*CfMSG5* complemented strain; Bar=10 μm. D. Significant difference in lesion diameter based on statistical analysis (**: P<0.01). E. Statistical analysis of the conidiation (2 days) of the wildtype strain (CFLH16), the Δ*Cfmsg5* mutant strain, and the ∆*Cfmsg5*/*CfMSG5* complemented strain (**: P<0.01). F. Statistical analysis of appressorium formation rate (24 h) of the wildtype strain (CFLH16), the Δ*Cfmsg5* mutant strain, and the ∆*Cfmsg5*/*CfMSG5* complemented strain (P>0.05). Error bars, SD. Data presented are the mean from three independent biological experiments.

Fig. 6 CfMsg5 plays an important role in *C. fructicola* pathogenicity, ROS clearance, and CWI MAPK cascade regulation

A. Conidial suspension (10^6^ conidia/ml) of CFLH16 and Δ*Cfmsg5* were sprayed on tea-oil leaves. CK: sterile water was sprayed on tea-oil leaves as a control. Diseased leaves were harvested 96 hours after inoculation; DAB staining after 24 hours and 96 hours. The black arrow is appressorium. Bar=25 μm.

B. H_2_O_2_ content were determined using visible spectrophotometry in leaves. Wavelength: 415nm.

C. The phosphorylation Level of Mpk1was determined using western blotting, before (-) and 30 min after addition of 5 mM H_2_O_2_ (+), using immunoblot analysis with antiphospho-p44/42 MAPK antibody (p-Mpk1). β-tubulin antibody was used as loading control. Ratio of pMpk1 versus β-tubulin levels, normalized to the WT strain at time 0. Error bars, SE. Data presented are the mean from three independent biological experiments.

Fig. 7 CfMsg5 regulates optimal growth, various stress response and unfolded protein response.

A: The growth rate of ∆*Cfmsg5* was significantly reduced in both CM and MM media in the dark for 3 days. B. Growth status of CFLH16, ∆*Cfmsg5* and ∆*Cfmsg5*/*CfMSG5* complemented strain in the dark for 3 days under 0.7M NaCl stress. C: Growth status of CFLH16, ∆*Cfmsg5* and ∆*Cfmsg5*/*CfMSG5* complemented strain on CM with 0/2.5/5/10 mM H_2_O_2_ at 28℃ in the dark for 3 days. D: Growth status of wildtype strain (CFLH16), ∆*Cfmsg5* mutant, and ∆*Cfmsg5*/*CfMSG5* complemented strains on CM supplemented with 0.5 μg/ml TUNI at 28℃ in the dark for 3 days. E: Growth status of wildtype strain (CFLH16), ∆*Cfmsg5* mutant, and ∆*Cfmsg5*/*CfMSG5* complemented strains on CM supplemented with 5 mM DTT at 28℃ in the dark for 2 days. F: Statistical analysis of diameter of strains colony in CM and MM (**: P<0.01; *: 0.05<P<0.01). G: Statistical analysis of growth inhibition rate of strains in different stress (**: P<0.01; *: 0.05<P<0.01). H: Expression level analysis of 6 selected genes in *C. fructicola*. Significant differences compared with the wildtype were estimated (**: P<0.01). Error bars, SD. Data presented are the mean from three independent biological experiments.

Fig. 8 Phosphatase activity of CfMsg5 is significant to pathogenicity and CWI MAPK cascade regulation.

A. The lesions caused by the CFLH16, the Δ*Cfmsg5* ^Ala461^ mutant strain, and the ∆*Cfmsg5*/*CfMSG5* complemented strain on wounded tea-oil leaves. B and G: The phosphorylation Level of Mpk1was determined using western blotting, before (-) and 30 min after addition of 5 mM H_2_O_2_ (+), using immunoblot analysis with antiphospho-p44/42 MAPK antibody (p-Mpk1). β-tubulin antibody was used as loading control. Ratio of pMpk1 versus β-tubulin levels, normalized to the WT strain at time 0; Error bars, SE (WT：CFLH16；∆：Δ*Cfmsg5* ^Ala461^ mutant; C：the ∆*Cfmsg5*/*CfMSG5* complemented strain). C: The appressorium of the CFLH16, the Δ*Cfmsg5* ^Ala461^ mutant strain, and the ∆*Cfmsg5*/*CfMSG5* complemented strain; Bar=20 μm. D: Significant difference in lesion diameter based on statistical analysis (**: P<0.01). E: Statistical analysis of the conidiation (2 days) of the CFLH16, the Δ*Cfmsg5* ^Ala461^ mutant strain, and the ∆*Cfmsg5*/*CfMSG5* (**: P<0.01). F: Statistical analysis of appressorium formation rate (24 h) of the CFLH16, the Δ*Cfmsg5* ^Ala461^ mutant strain, and the ∆*Cfmsg5*/*CfMSG5* (P>0.05). Error bars, SD. Data presented are the mean from three independent biological experiments.

Fig. 9 Phosphatase activity of CfMsg5 is important to stress response

A: The growth rate of ∆*Cfmsg5* ^Ala461^ was significantly reduced in MM media in the dark for 4 days. B. Growth status of CFLH16, ∆*Cfmsg5* and ∆*Cfmsg5*/*CfMSG5* complemented strain in the dark for 3 days under 0/2.5/5/10 mM H_2_O_2_ stress or 5 mM DTT. C: Statistical analysis of diameter of strains colony in CM and MM (**: P<0.01). D: Statistical analysis of growth inhibition rate of strains in different stress (**: P<0.01). Error bars, SD. Data presented are the mean from three independent biological experiments.

**Supporting Information**

Fig. S1. Domain Prediction and Phylogenetic Analysis of CfAp1.

A. The domain architecture of CfAp1 was predicted using the Simple Modular Architecture Research Tool (SMART) website (http://smart.embl-heidelberg.de/). The basic-leucine zipper domain is denoted as BRLZ, and the numbers indicate the amino acid residue positions. pink boxes represent an unknown domain. B. The phylogenetic tree was constructed using the neighbour-joining (NJ) method and analysed with 1,000 bootstrap replicates in MEGA7. The numbers on the branches represent the bootstrap support calculated with 100 replicates in the NJ analysis, and the scale bar indicates the branch length. The GenBank accession numbers of the amino acid sequences orthologous to CfAp1 are shown before each reference taxon.

Fig. S2 GO enrichment analysis of genes in module “turquoise”.

Fig. S3 redox related genes expression during Cf infection.

Fig. S4 others. A: Primer1: MSG5-5F/H855R; Primer2: MSG5-7F/ MSG5-8R; M: DL5000 marker; -: H_2_O negative control; +: WT positive control; Δ1: the *CfMSG5* gene deletion mutant(∆*Cfmsg5*); Δ2: A mutant that changed the CfMsg5 for Cys461 to Ala461(∆*Cfmsg5* ^Ala461^). B: Appressorium of CFLH16, ∆*Cfap1* and complemented strain, Bar=20 μm. C. Level of expression of A12032, A10759 and A15105 in ∆*Cfap1*. D: A12032, A10759 and A15105 frontal spots on fresh *Camellia oleifera* leaves. (-∆: mutant strains, -C: complemented strains.).

Fig. S5 redox related genes expression during Cf infection in *ΔCfAP1.*
